# Investigating Coriander Leaf Phenolics With HPLC‐UV and Their Role in Modulating Nitrogen Metabolism

**DOI:** 10.1002/fsn3.70029

**Published:** 2025-03-17

**Authors:** Mahr‐Un Nisa, Muhammad Umer, Muhammad Hamza, Huma Umbreen, Nukhba Khalid, Muhammad Qasim Raza, Isam A. Mohamed Ahmed, Moneera O. Aljobair, Osman Ahmad Khan

**Affiliations:** ^1^ Department of Nutritional Sciences, Faculty of Medical Sciences Government College University Faisalabad Punjab Pakistan; ^2^ Faculty of Pharmaceutical Sciences Government College University Faisalabad Punjab Pakistan; ^3^ Punjab Food Authority Lahore Lahore Punjab Pakistan; ^4^ Department of Food Science and Nutrition, College of Food and Agricultural Sciences King Saud University Riyadh Saudi Arabia; ^5^ Department of Sports Health, College of Sports Sciences & Physical Activity Princess Nourah Bint Abdulrahman University Riyadh Saudi Arabia; ^6^ Livestock and Dairy Development Department Faisalabad Punjab Pakistan

**Keywords:** biochemical parameters, coriander ( *Coriandrum sativum* ), HPLC‐UV, nitrogen balance, nutrient digestibility, quercetin, uric acid, weight management

## Abstract

Coriander leaves (*Coriandrum Sativum L.*) contain quercetin, a flavanol from the flavonoid polyphenol group that helps prevent various metabolic disorders. This study aimed to use HPLC‐UV to investigate phenolic compounds in coriander leaves and their impact on nitrogen metabolism. Twenty‐four healthy Wistar albino rats weighing 160 ± 10 g were divided into four groups: NC (no coriander), CT_1_, CT_2_, and CT_3_. They received coriander leaf powder at levels of 12.2, 16, and 20.33 g/100 g of feed, respectively, based on the availability of quercetin on a dry matter (DM) basis. Feed intake was found higher in CT_3_ (27.27 g) and CT_2_ (25.43 g), while CT_1_ (24.96 g) and NC (24.66 g) showed a nonsignificant (*p* ≥ 0.05) trend; however, a similar trend was observed in weekly feed intake. After three weeks, rats in the CT_3_ group showed a 20 g reduction in body weight compared to the NC group (149.64 g vs. 163.41 g). The feed conversion and feed efficiency ratios (FCR & FER) were decreased in all treated groups due to the reduction in body weight (BW). The digestibility of DM and soluble carbohydrates was reduced in rats fed the CT_3_ diet while crude protein (CP), crude fiber (CF), ether extract (EE), and ash showed increasing trends. The rats that were fed different levels of coriander in the diet showed positive nitrogen balance. There was a reduction in serum uric acid in CT_1_ (0.72 ± 0.04 mg/dL) and CT_2_ (0.81 ± 0.03 mg/dL) as compared to other treatment groups. These nitrogen parameters had a positive impact on nitrogen metabolism, along with a significant (*p* ≤ 0.05) effect on total serum protein and a non‐significant (*p* ≥ 0.05) effect on creatinine. Liver enzymes showed significant improvements in rats that were fed varying amounts of coriander powder. High‐density lipoprotein (HDL) levels improved in all treatment groups, with a significant increase observed in CT_1_ (52.27 ± 0.27 mg/dL). In conclusion, 130 g of dry coriander containing 136 mg of quercetin can be effectively included in various food recipes in dry or wet form to improve serum indices and weight management in healthy people.

## Graphical abstract

Coriander has the antibacterial, immune‐boosting, anti‐inflammatory, free radical scavenging, antilipidemic, and antiaging it significantly lower the prevalence of hyperuricemia. Coriander inhibits the xanthine oxidase enzyme because of the variable amount of quercetin that has the capacity to reduce uric acid in the body. Therefore, in this research study, Coriander leaf powder is used to prevent the formation of uric acid crystals, which proved to be much more effective and have no side effects.

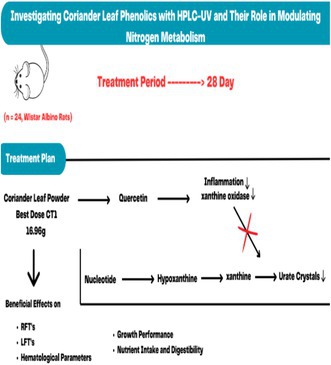

**Abbreviations**
ACC: Acetyl‐CoA carboxylase
ALP: Alkaline phosphatase
ALT: ALANINE aminotransferase
AST: Aspartate Aminotransferase
BUN: Blood urea nitrogen
BW: Body weight
CF: Crude fiber
CP: Crude protein
CT1: Coriander treatment 1
CT2: Coriander treatment 2
CT3: Coriander treatment 3
DGAT: Diacylglycerol acyltransferase
DM: Dry matter
EDTA: Ethylenediaminetetraacetate
EE: Ether extract
FCR: Feed conversion ratio
FER: Feed efficiency ratio
Hb: Hemoglobin
HDL: High‐density lipoprotein
HPLC: High performance liquid chromatography
LDL: Low‐density lipid
NC: Normal control
NFE: Nitrogen free extract
RBCs: Red blood cells
SCFAs: Short‐chain fatty acids
TAA: Thioacetamide
VLDL: Very low‐density lipoprotein
WBCs: White blood cells

## 1 Introduction

Due to its appealing green color and flavor, coriander ( *Coriandrum sativum* L.) leaves are most widely used in Asia, India, Central Europe, and North Africa for various recipes (sauces, salads, and garnish‐cooked food). Due to the recent emergence of nutraceutical components, such as chlorogenic acid, vanillic acid, caffeic acid, ferulic acid, p‐coumaric acid, quercetin, kaempferol, and apigenin, it is also utilized as a medical plant. One of these is quercetin, which has a higher concentration in coriander than in other flavonoids in the same family (Kozłowska et al. 2021). The leaves from different regions are utilized as flavoring agents and condiments, and they show promising effects on various disorders, including indigestion, abdominal distention, diarrhea, urinary tract infections, and asthma. Additionally, these leaves have been used to alleviate nausea and vomiting (Scandar, Zadra, and Marcotullio 2023). Coriander contains a suitable amount of quercetin, as well as optimal levels of fiber, protein, minerals, ascorbic acid, and vitamin A. This combination of nutrients makes it a valuable addition to various dishes for home cooking. Quercetin plays an important role in several body functions. People often use coriander leaves and seed powder in their meals because it adds a favorable taste. Various studies have been conducted to explore the nutritional values of coriander leaves, seeds, and flowers (Frydrych, Krośniak, and Jurowski 2023; Bhat et al. 2014). Along with having antibacterial, immune‐boosting, anti‐inflammatory, free radical scavenging, antilipidemic, antiaging, and anticancer actions, it is crucial for sustaining growth performance and digestion (Abdel‐Latif et al. 2021; David et al. 2016; Mechchate et al. 2021). According to various academic studies, it also significantly lowers the prevalence of hyperuricemia. The metabolic by‐product of purine and pyrimidine nucleic acids is uric acid. Reactive oxygen species are produced as xanthine dehydrogenase/xanthine oxidase (XDH/XO) catabolizes the formation of uric acid, increasing the risk of high blood pressure, elevated cholesterol levels, diabetes, and atherosclerosis (Punzi 2022). The main contributors to increased uric acid in the diet are purine and fructose intake (Su et al. 2020). A naturally occurring sugar molecule called fructose speeds up the metabolism of de novo purines by lowering ATP levels, which causes a rise in blood uric acid levels. High fructose consumption, such as white sugar in tea and carbonated beverages, is becoming a bad habit among Pakistanis, and per capita sugar consumption has increased by up to 24 kg from 2018–19. The average person consumes 16 tablespoons of sugar each day, which accounts for around 3.5% of all food expenses in the kitchen (Lubawy and Formanowicz 2023). Pure fructose is found in various products, such as high fructose corn syrup, sweets, and processed foods. It plays a significant role in increasing the production of uric acid in the body. Daily fructose consumption of 25–50 g is considered safe, while 50–100 g is considered high but manageable (Chhapra et al. 2010). However, an intake exceeding 100 g per day is considered dangerous for the human body (Aoun et al. 2022; Caliceti et al. 2017). Because it has been demonstrated in earlier research that coriander inhibits the xanthine oxidase enzyme, it has a variable amount of quercetin and the capacity to reduce uric acid in the body (Mo et al. 2007; Hu et al. 2009). The study was designed to introduce a new, economical, and indigenous source of organic compounds, along with a food‐based strategy to reduce the incidence of hyperuricemia, emphasizing that prevention is better than cure. The other objective is to explore the nutraceutical perspective of coriander powder through rat modeling. Currently, the main therapeutic approaches for treating hyperuricemia involve the use of allopurinol and febuxostat, which are associated with various side effects. Therefore, there is a need to quantify the quercetin content in natural sources and to address this issue by introducing appropriate levels of various functional compounds. Previous studies have demonstrated that coriander exhibits inhibitory activity against xanthine oxidase in vitro (Kim 2023). This study aims to evaluate the efficacy of coriander in rats. The rats were given different levels of coriander, corresponding to varying amounts of quercetin, as there is a research gap in previous studies regarding this topic. The goal is to identify the optimal level of coriander leaf powder that provides a safe amount of quercetin. Additionally, this study will determine the optimum levels of coriander, both in their raw forms and on a dry matter basis, using the human equivalent dose formula for accurate assessment. As a part of this, this study also aimed to assess the impact of different levels of coriander leaf powder, corresponding to the level of quercetin, on modulating nitrogen metabolism and biochemical and hematological traits in healthy male albino rats.

## 2 Materials and Methods

### 2.1 Procurement of Materials and Chemicals

**FIGURE 1:**
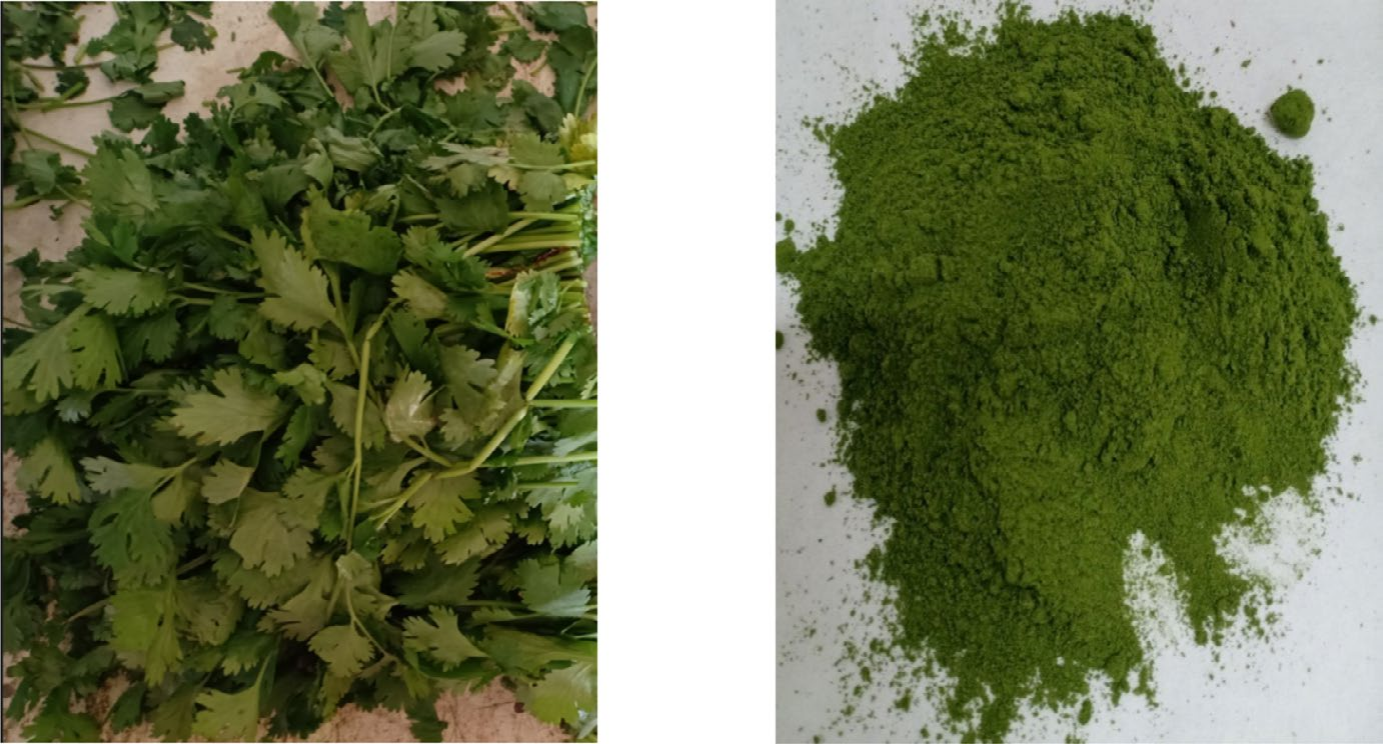
Processing and preparation of coriander powder for the supplementation and HPLC‐UV analysis. Preparation of the coriander leaf powder for further analysis by HPLC‐UV to confirm the presence of quercetin.

The healthy and highly seasoned coriander was purchased from the market after being cleaned of any contaminants; they were dried in a hot air (BIOBASE HAS‐T105 China) oven at 65°C. Using an electric lab grinder (NIMA NM‐8300 Japan), dried coriander is ground into a fine powder and stored in plastic bags at 4°C (Figure 1) as described by Hussain et al. (2022), AOAC (2006). All chemicals of analytical grade were purchased from Sigma‐Aldrich in St. Louis, Missouri, USA. Analysis kits were procured from scientific stores and local vendors in Faisalabad, Pakistan.

### 2.2 Quercetin Determination in Coriander Leaf Powder Using HPLC‐UV

#### 2.2.1 Preparation of Sample

Coriander leaf powder (10 g) was incorporated with methanol and water in an 80:20 volumetric ratio to prepare coriander extract. After being shaken at 150 rpm for 4 h, it was filtered using Whatman filter paper. The yield extract was once more extracted using the same solvent ratio, and methanol was then evaporated using a rotary evaporator (BIOLAND RE‐5000A China) at 40°C. The final semisolid extract was collected and shifted for HPLC‐UV analysis (Pandey et al. 2011). The Department of Botany at Government College University in Faisalabad provided the standard for quercetin.

#### 2.2.2 Nutrient Composition of Coriander Leaf Powder and HPLC‐UV Method

The nutrient composition of coriander leaf powder was analyzed using the specified method (AOAC 2006; Rafique et al. 2023). Chemicals for HPLC were purchased from Sigma‐Aldrich in St. Louis, Missouri, USA. The most recent Agilent HP1200 was intended to be assigned with a G1322A quaternary pump and degasser, a G1314A wavelength UV detector, and a particle size remained at 5 m (250 4.0 mm) ODS column (Lichrospher 100, Merck, Darmstadt, Germany) for the identification and quantification of quercetin in methanol extract of coriander. 1 mL sample was added to a 50 mL volume in HPLC‐grade water. After that, the sample was filtered using Whatman No. 1 filter media with a 25 m pore size for 5 min in a water bath shaker at 30°C. A mobile phase of HPLC‐grade water was used for analysis after the prepared sample was inoculated into the HPLC apparatus. The results were recorded while maintaining a flow rate of 1 mL/min, and the examined components in the yield extract were recorded by comparing them to the reference standard of quercetin and kaempferol. A linear gradient system using mobile phase A (95% formic acid in water by volume) and mobile phase B (methanol) was used to achieve chromatographic separation. 50% mobile phase B for 10 min, rising to 80% B over 10 min, then to 100% B over 11 min, and then returning to B at 50%, finished the linear gradient elution. UV observation was detected at 360 nm at a flow rate of 1.0 mL/min (Rahim et al. 2022).

### 2.3 Composition of Diet

Following (NRC 2001), *isocaloric* and *isonitrogenous* diets were prepared, and the vitamins and minerals were incorporated as described in Table 1. The diet was planned to have 3766 kcal/kg of calories and be distributed as follows: 19.3% protein, 64.0% carbohydrates, and 16.7% fat. The diet was prepared daily according to the requirements of the animals (Reeves, Nielsen, and Fahey 1993).

**TABLE 1:** Nutrient composition of diet (g/kg).

Groups				
Ingredients	NC	CT_1_	CT_2_	CT_3_
Corn starch	286	286	286	286
Dextrose	203	203	203	203
Corn oil/soybean oil	70	70	70	70
Soyabean meal	396	396	396	396
AIN‐93‐MX mineral mix	35	35	35	35
AIN‐93‐vitamin mix	10	10	10	10
TBHQ (Tert‐butylhydroquinone)	0.008	0.008	0.008	0.008
Total calories (kcal/kg)	3266	3266	3266	3266
Coriander (g)		**12.2 g (0.75 mg/kg)**	**16.26 g (0.100 mg/kg)**	**20.33 g (0.125 mg/kg)**
**Nutritive value %**				
Dry matter	79 ± 0.14	83 ± 0.12	84.2 ± 0.15	84.9 ± 0.08
Crude protein	18.63 ± 0.27	18.32 ± 0.19	18.25 ± 0.15	18.16 ± 0.11
Crude fiber	1.27 ± 0.09	1.49 ± 0.08	1.59 ± 0.10	1.74 ± 0.07
Crude fat	6.81 ± 0.11	6.43 ± 0.10	6.40 ± 0.15	6.32 ± 0.08
Ash	3.70 ± 0.13	4.32 ± 0.10	4.50 ± 0.11	4.57 ± 0.9
Moisture	21 ± 0.13	17 ± 0.11	15.8 ± 0.14	15.1 ± 0.13
NFE	49.59 ± 0.12	52.44 ± 0.11	53.46 ± 0.14	54.11 ± 0.15

*Note:* NC, normal control with 0 g Coriander leaf powder, Coriander Treatment 1 (CT_1_) with 12.2 g/100 g coriander leaf powder, Coriander Treatment 2 (CT_2_) with 16.26 g/100 g coriander leaf powder and Coriander Treatment 3 (CT_3_) with 20.33 g/100 g coriander leaves powder. Data was analyzed by using one‐way ANOVA, followed by multiple comparisons using LSD, and data was considered significant at *p* ≤ 0.05*. *p* ≤ 0.05 means significant and *p* ≥ 0.05 means results are non‐significant. The levels were formulated on the basis of quercetin present in the coriander which means the quercetin rich coriander contains 0.075mg and 0.100 and 0.125mg/kg quercetin in 12.2g/100g and 16.26g/100g and 20.33g/100g of coriander leaf powder. Thse levels were offered to the two replicates of the 1 group. After addition of these levels in the coriander the nutritive value of diets were determined which is present at the end of the coriander. The diet was selected isocaloric and isonitrogenous. This signifies the important to evaluate intake, weight managment, serum, hematological and nitrogen balance parameters in male healthy wistar albino rats. This study might be important for nutritonist and researchers and health care professionals.

### 2.4 Animals and Study Design

The study was conducted in collaboration with the Department of Nutritional Sciences and the Department of Physiology. All the experimental techniques were carried out under rules and guidelines for the safe use of laboratory animals. Vide notification No. GCUF/ERC/85, the Animal Ethical Committee of the Government College University Faisalabad, Pakistan, had approved conducting trials. A research study was conducted on 24 healthy male Wistar albino rats weighing 160 ± 10 g in an animal home with a humidity of 45%–55% and a temperature of 25°C (Wade 1978). Rats were divided into four groups randomly, with six rats per group. Coriander leaf powder was given to the NC with no coriander, CT_1_, CT_2_, and CT_3_ groups at levels of 12.2, 16.26, and 20.33 g/100 g feed corresponding to the levels of quercetin 0.075, 0.100, and 0.125 mg/kg, respectively. The groups of rats were divided into two subgroups, each subgroup containing three rats. The main purpose of this is to control the rats ethically, and each subgroup offered 100 g of feed.

### 2.5 Evaluation of Growth Performance and Nutrient Digestibility

Throughout the trial, animals were offered food and water ad libitum. The study duration lasted 42 days, with 14 days of acclimatization and 28 days of treatment and collection period. To determine the amount of feed and nutrients consumed on a daily and weekly basis, the feed offered, consumed, and leftovers were recorded. The difference between the amount of nutrients consumed and eliminated in feces was used to calculate the nutrient digestibility. The last 7 days were taken into consideration when collecting feces, and the dry matter (DM) of the feces was calculated by putting a sample in a hot air oven at 60°C for 72 h. The DM of the meal sample was similarly calculated by this method in the same manner. The formulas for calculating nutrient digestibility, feed conversion ratio, and feed efficiency ratio are given below (Umer et al. 2023, 2024; Shi et al. 2015; Nisa et al. 2006).

(1) $$\mathrm{FCR}\;\left(\%\right)=\frac{\text{Feed Intake}}{\text{Body Weight}}$$

(2) $$\mathrm{FER}=\frac{\text{Body weight}}{\text{Feed Intake}}$$

(3) $$\text{Digestibility}=\frac{\text{Nutrient Intake}-\text{Nutrient in feces}}{\text{Nutrient Intake}}\times 100$$

### 2.6 Blood Collection

All of the rats were anesthetized and decapitated at the end of the experiment to assess the outcome. Blood was drawn from the neck area using a surgical blade, and after collection, immediately preserved in ethylenediaminetetraacetate (EDTA) tubes. Following 5000 rpm centrifugation, serum was kept at −20°C for various biochemical analyses as described by Hussain, Arif, et al. (2024) and Hussain, Korma, et al. (2024).

### 2.7 Biochemical Analysis

Aspartate aminotransferase (AST) (U/L), alanine aminotransferase (ALT) (U/L), alkaline phosphatase (ALP) (U/L), serum bilirubin (mg/dL), total protein (g/dL), creatinine (mg/dL), blood urea nitrogen (BUN), and uric acid (mg/dL) were also measured using commercially available kits. These measurements were made using the Cobas E311 method, which is based on the principle of a spectrophotometer (Randie 2016). In an investigation of lipid profile, serum total cholesterol (TC) (mg/dL), triglycerides (TG) (mg/dL), high‐density lipoprotein (HDL) (mg/dL), and low‐density lipoprotein (LDL) (mg/dL) were estimated by commercially available kits. Kit for uric acid (CAT no. UA 121120) BioMed Diagnostics was provided by the Nutritional Sciences Department of Government College University, Faisalabad. Hematological parameters, including hemoglobin (Hb) (g/dL), red blood cell count (RBC) (×10^6^ μL), white blood cell count (WBC) (×10^3^ μL), monocytes, lymphocytes, neutrophils (%), and platelet count (×10^5^ μL), were analyzed by use of Mindray BC‐6200 based on the principle of electrical impedance, flow cytometry, and light scattering (Kulik et al. 2021).

### 2.8 Nitrogen Balance Study

Animals were transferred to metabolic cages at the start of the collection period, and nitrogen‐free Whatman filter paper was pre‐weighed and placed underneath the cages by sliding them on aluminum foil. After 24 h, papers were collected, re‐weighed, and kept in various plastic zip‐lock bags for more analysis. Each paper's N% was determined using the standard method of AOAC (Kumar et al. 2021; NRC 2001; AOAC 2006). Nitrogen balance was estimated by the following equation

(4) $$\text{Nitrogen Balance}=\%\text{Nitrogen}\;\left(\text{Feed}-\text{Urine}-\text{Feces}\right)$$

### 2.9 Statistical Analysis

Using statistics software 8.1 Steel (1997) all of the data from the findings were statistically assessed for mean and standard deviation as well as one‐ and two‐way ANOVA. If the “*p*” value was close to or less than 0.05 or larger than 0.05, the difference in mean values between the groups was considered significant; otherwise, it was non‐significant.

### 2.10 Human Equivalent Dose

According to the present study, the human equivalent dose of coriander leaf is 130.07 g with quercetin 136 mg on such a basis, while on a dry matter basis, it is 16.96 g and quercetin 156 mg (Shin, Seol, and Son 2010). The following equation was used to calculate the human equivalent dose.

(5) $$\text{Human Equivalet Dose}\left(\frac{\mathrm{mg}}{\mathrm{kg}}\right)=\text{Animal dose}\times \text{Conversion factor}$$

## 3 Results

### 3.1 Nutrient Composition and Quantification of Coriander Leaf Powder Using HPLC‐UV

**FIGURE 2:**
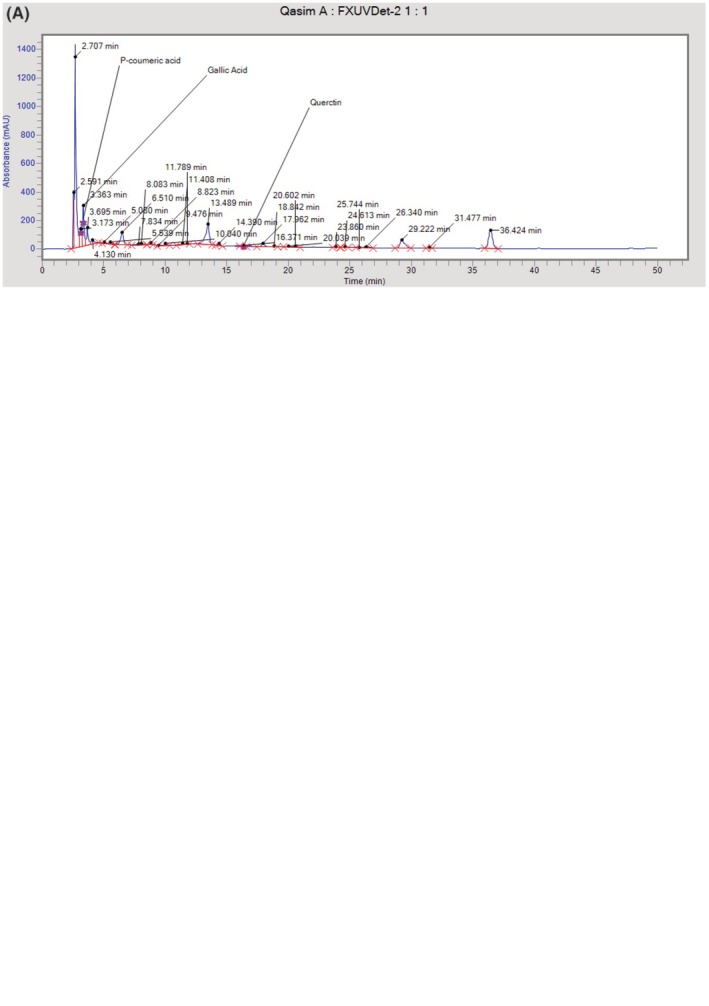
(A) Quantification of quercetin present in coriander leaf powder using HPLC‐UV. A graph showing the quantification of quercetin in coriander leaf powder analyzed by HPLC‐UV. (B) Standard of quercetin (Umer et al. 2023, 2024). A graph showing the standard of quercetin by HPLC‐UV.

The nutritional evaluation of coriander leaf in this study revealed its moisture content of 86.96%, crude protein (CP) (15.31%), ether extract (EE) (0.89%), crude fiber (CF) (6.02%), ash (1.8%), and nitrogen‐free extract (NFE) (10.98%). The coriander leaf powder HPLC analysis revealed a main peak with a major peak area of 135,267.5, a height of 93722.2, and a retention time *t*_R_ of 16.371 at 360 nm. Compared to the quercetin standard, which has a peak area of 2,545,071.5, height of 280,423.6, and retention time *t*_*R*_ of 3.363 at 360 nm. The study's findings indicate that coriander has 18.82 mg of quercetin per 100 g of dry weight, whereas the standard for quercetin had a *t*_*R*_ value of 16.954 at 360 nm absorption. The retention of an analyte on a chromatographic column is determined using the accepted *K* factor, commonly referred to as a capacity factor, which is 0.000696. The chromatogram and peaks of the standard and coriander leaf HPLC‐UV data are shown in Figure 2A,B.

### 3.2 Effect of Coriander on Growth Performance

A trend in feed intake was found to be higher both in treatment and weeks. Treatment‐wise feed intake was found higher (*p* ≤ 0.05) in CT_3_ (27.27 g) and CT_2_ (25.43 g) in comparison with CT_1_ (24.96 g) and NC (24.66 g), which showed a nonsignificant trend (*p* ≥ 0.05). Weekly feed intake was found higher in week 2 (26.96 g) and week 3 (27.60 g) in comparison with week 1 (22.61 g). The week‐wise mean value of body weight (BW) that was significantly (*p* ≤ 0.05) decreased in the first week and seemed to simultaneously catch up in average BW in the next two weeks. Treatment‐wise, the mean value of BW was significantly (*p* ≤ 0.05) found to be higher (163.41 g) and lower (149.64 g) in rats fed NC and CT_3_ diets than those fed other treatment diets. Water intake was consistent within the treatment groups, but a negligible decrease was observed in CT_3_ (23.89 mL) compared with the NC (24.57 mL). Weekly water intake was increased in week 2 (25.08 mL) and week 3 (27.16 mL) in comparison with week 1 (21.35 mL). Feed conversion ratio (FCR) and feed efficiency ratio (FER) values were significantly decreased (*p* ≤ 0.05) among the coriander‐treated diets than those fed NC diets because there is a significant difference in initial and final body weight (Table 2).

**TABLE 2:** Effect of coriander on growth performance.

Groups	Weekly growth performance				
	Week 1		Week 2	Week 3	Mean
**Feed Intake (g)**					
NC	24.15 ± 0.22		25.26 ± 0.20	24.59 ± 0.16	24.66^C^
CT_1_	22.00 ± 0.19		26.08 ± 0.25	26.81 ± 0.16	24.96^C^
CT_2_	21.69 ± 0.14		27.80 ± 0.14	28.54 ± 0.21	25.43^B^
CT_3_	22.63 ± 0.17		28.70 ± 0.22	30.49 ± 0.14	27.27^A^
Mean	22.61^C^		26.96^B^	27.60^A^	
**Water Intake (mL)**					
NC	22.96 ± 2.91		24.13 ± 3.56	26.64 ± 3.45	24.57^AB^
CT_1_	22.00 ± 3.31		26.30 ± 1.76	26.61 ± 2.63	24.97^A^
CT_2_	21.41 ± 2.56		25.64 ± 3.02	27.04 ± 2.55	24.69^A^
CT_3_	19.04 ± 1.59		24.27 ± 2.88	28.38 ± 2.60	23.89^B^
Mean	21.35^C^		25.08^B^	27.16^A^	
**Body weight (g)**					
	**Week 0**	**Week 1**	**Week 2**	**Week 3**	**Mean**
NC	168.47 ± 6.23	143.80 ± 0.16	173.05 ± 1.38	168.32 ± 1.53	163.41^A^
CT_1_	161.21 ± 0.82	146.71 ± 0.62	145.39 ± 0.35	150.45 ± 0.22	150.94^C^
CT_2_	161.19 ± 0.79	146.81 ± 0.63	154.34 ± 0.46	160.25 ± 0.19	155.65^B^
CT_3_	161.29 ± 0.56	145.05 ± 0.16	144.47 ± 0.48	147.76 ± 0.12	149.64^C^
Mean	163.04^A^	145.59^D^	154.31^C^	156.70^B^	
**Feed conversion ratio (FCR) and feed efficiency ratio (FER)**					
**Groups**					
**Parameters**	**NC**	**CT**_**1**_	**CT**_**2**_	**CT**_**3**_	
FCR (%)	164.4 ± 0.15^a^	−2.31 ± 0.11^d^	27.05 ± 0.28^b^	−2.01 ± 0.16^d^	
FER	0.006 ± 0.13^b^	−0.431 ± 0.09^d^	0.036 ± 0.04^a^	−0.496 ± 0.08^d^	

*Note:* NC, normal control with 0 g Coriander leaf powder, Coriander Treatment 1 (CT_1_) with 12.2 g/100 g coriander leaf powder, Coriander Treatment 2 (CT_2_) with 16.26 g/100 g coriander leaf powder and Coriander Treatment 3 (CT_3_) with 20.33 g/100 g coriander leaves powder. Data was analyzed by using two‐way ANOVA, followed by multiple comparisons using LSD and data was considered significant at *p* ≤ 0.05*. *p* ≤ 0.05 means significant, and *p* ≥ 0.05 means results are non‐significant. FCR and FER values were analyzed by using one‐way ANOVA. Mean within the same column with different superscripts (a–d) is statistically different at *p* ≤ 0.05.

Abbreviations: FCR, feed conversion ratio; FER, feed efficiency ratio.

### 3.3 Effect of Coriander on Nutrient Intake

In this study, the ratio of feed intake and nutrient digestibility is directly related to the ratio of nutrient intake in rats. A significant positive trend (*p* ≤ 0.05) was seen in DM, CP, CF, EE, Ash, and NFE intake in all treatment groups. Rats offered a coriander‐enriched diet CT_3_ had been shown to have increased in all nutrient intake, followed by the better intake observed in the CT_2_ group (Table 3).

**TABLE 3:** Effect of coriander on nutrient intake.

Nutrient %	Groups			
	NC	CT_1_	CT_2_	CT_3_
Dry matter	19.42 ± 0.28^d^	22.25 ± 0.32^c^	24.03 ± 0.23^b^	25.88 ± 0.23^a^
Crude protein	4.58 ± 0.09^b^	4.91 ± 0.45^b^	5.20 ± 0.20^a^	5.53 ± 0.11^a^
Crude fiber	0.31 ± 0.07^c^	0.39 ± 0.08^ab^	0.45 ± 0.04^a^	0.53 ± 0.05^a^
Ether extract	1.67 ± 0.11^c^	1.72 ± 0.09^ab^	1.82 ± 0.07^a^	1.80 ± 0.08^a^
Ash	0.90 ± 0.08^d^	1.15 ± 0.46^c^	1.28 ± 0.07^ab^	1.39 ± 0.17^a^
NFE	11.96 ± 0.12^c^	16.83 ± 0.28^b^	15.28 ± 0.12^b^	19.63 ± 0.12^a^

*Note:* NC, normal control with 0 g Coriander leaf powder, Coriander Treatment 1 (CT_1_) with 12.2 g/100 g coriander leaf powder, Coriander Treatment 2 (CT_2_) with 16.26 g/100 g coriander leaf powder, and Coriander Treatment 3 (CT_3_) with 20.33 g/100 g coriander leaves powder. Data was analyzed by using one‐way ANOVA, followed by multiple comparisons using LSD, and data was considered significant at *p* ≤ 0.05*. *p* ≤ 0.05 means significant, and *p* ≥ 0.05 means results are non‐significant. Mean within the same column with different superscripts (a–d) is statistically different at *p* ≤ 0.05.

Abbreviation: NFE, nitrogen free extract.

### 3.4 Effect of Coriander on Nutrient Digestibility

DM digestibility was significantly decreased (*p* ≤ 0.05) (76.35% ± 0.91%), (76.54% ± 0.34%) in rats fed CT_1_ and CT_3_ diets and higher in CT_2_ (80.81% ± 0.73%), which is non‐significant (*p* ≥ 0.05) to the NC (81.10% ± 0.66%). CP was significantly (*p* ≤ 0.05) increased in the coriander‐treated diets but higher (86.79% ± 0.28%) and (83.23% ± 0.39%) in rats fed CT_3_ and CT_2_ diets as compared to the NC (82.30% ± 0.31%). Crude fiber digestibility was found higher (84.67% ± 0.20%) in rats offered CT_3_ diet and other treatment diets did not show an improvement in comparison with the NC (83.48% ± 0.31%) as shown (Table 4). EE digestibility was found higher (86.32% ± 0.29%) and lower (80.42% ± 0.35%) in rats fed CT_3_ and CT_2_ diets in comparison with the CT_1_ (82.63% ± 0.19%) and NC (82.81% ± 0.24%) diets. Digestibility of ash significantly increased (*p* ≤ 0.05) in CT_3_ (83.65% ± 0.26%), while other coriander‐treated groups showed non‐significant (*p* ≥ 0.05) results in comparison with NC.

**TABLE 4:** Effect of coriander on nutrient digestibility.

Nutrient %	Groups			
	NC	CT_1_	CT_2_	CT_3_
Dry matter	81.10 ± 0.66^a^	76.35 ± 0.91^b^	80.81 ± 0.73^a^	76.54 ± 0.34^b^
Crude protein	82.30 ± 0.31^c^	82.74 ± 0.25^c^	83.23 ± 0.39^b^	86.79 ± 0.28^a^
Crude fat	83.48 ± 0.31^b^	80.19 ± 0.39^c^	80.11 ± 0.27^c^	84.67 ± 0.20^a^
Ether extract	82.81 ± 0.24^b^	82.63 ± 0.19^b^	80.42 ± 0.35^c^	86.32 ± 0.29^a^
Ash	80.59 ± 0.29^b^	79.60 ± 0.26^b^	78.08 ± 0.31^c^	83.6 5 ± 0.26^a^
NFE	39.23 ± 0.29^a^	33.10 ± 0.39^b^	33.85 ± 0.20^b^	29.39 ± 0.31^d^

*Note:* NC, normal control with 0 g Coriander leaf powder, Coriander Treatment 1 (CT_1_) with 12.2 g/100 g coriander leaf powder, Coriander Treatment 2 (CT_2_) with 16.26 g/100 g coriander leaf powder, and Coriander Treatment 3 (CT_3_) with 20.33 g/100 g coriander leaves powder. Nitrogen Free Extract (NFE), Data was analyzed by using one‐way ANOVA, followed by multiple comparisons using LSD, and data was considered significant at *p* ≤ 0.05*. *p* ≤ 0.05 means significant, and *p* ≥ 0.05 means results are non‐significant. Mean within the same column with different superscripts (a–d) is statistically different at *p* ≤ 0.05.

### 3.5 Effect of Coriander on Nitrogen Balance

In Table 5, more nitrogen intake was consistent with the increase in intake of CP and its digestibility. In CT_2_ nitrogen intake (0.39 ± 0.16 mg/day) was succeeded by its excretion in urine (0.01 ± 0.14 mg/day) and feces (0.28 ± 0.12 mg/day) in comparison with the other treatment groups, which showed negative nitrogen balance means more nitrogen excretion succeeded, then the nitrogen intake. In CT_1_, CT_2_, and NC, the intake of CP and digestibility was significantly improved (*p* ≤ 0.05) when compared to CT_2_; however, these treatment diets did not show appreciable nitrogen retention.

**TABLE 5:** Effect of coriander on nitrogen balance.

Parameters, mg/day	Groups			
	NC	CT_1_	CT_2_	CT_3_
Nitrogen intake	0.43 ± 0.02^a^	0.40 ± 0.26^b^	0.39 ± 0.16^c^	0.44 ± 0.18^a^
Urinary nitrogen	0.02 ± 0.006^a^	0.05 ± 0.12^b^	0.01 ± 0.14^c^	0.05 ± 0.20^b^
Fecal nitrogen	0.34 ± 0.008^a^	0.31 ± 0.11^b^	0.28 ± 0.12^d^	0.33 ± 0.10^a^
Nitrogen balance	0.07 ± 0.009^a^	0.03 ± 0.07^c^	0.09 ± 0.10^a^	0.05 ± 0.12^b^

*Note:* NC, normal control with 0 g Coriander leaf powder, Coriander Treatment 1 (CT_1_) with 12.2 g/100 g coriander leaf powder, Coriander Treatment 2 (CT_2_) with 16.26 g/100 g coriander leaf powder, and Coriander Treatment 3 (CT_3_) with 20.33 g/100 g coriander leaves powder. Data was analyzed by using one‐way ANOVA, followed by multiple comparisons using LSD, and data was considered significant at *p* ≤ 0.05*. *p* ≤ 0.05 means significant, and *p* ≥ 0.05 means results are non‐significant. Mean within the same column with different superscripts (a–d) is statistically different at *p* ≤ 0.05.

### 3.6 Effect of Coriander on Liver Function and Serum Total Protein

Rats offered a coriander diet had shown a significantly improved effect (*p* ≤ 0.05) on serum biochemical parameters, including liver, renal, total protein, and lipid profile. Serum ALT, AST, and ALP were significantly decreased (*p* ≤ 0.05) by (30.3 ± 0.27 U/L), (229 ± 0.57 U/L), and (216 ± 4.99 U/L) in rats fed coriander‐added diets CT_3_ and CT_1_ in comparison with the NC. Similarly significant decreases (0.06 ± 0.004 mg/dL), (0.01 ± 0.03 mg/dL) (*p* ≤ 0.05) in total bilirubin were observed in rats fed CT_2_ and CT_3_ diets as compared to the NC (0.06 ± 0.002 mg/dL). The status of total protein was also significantly improved (*p* ≤ 0.05), and a higher improvement (7.04 ± 0.03 g/dL) was seen in treatment level CT_3_ (Table 6).

**TABLE 6:** Effect of coriander on liver and renal function, serum total protein, and lipid profile.

Parameters	Groups			
	NC	CT_1_	CT_2_	CT_3_
**Liver function**				
ALT (U/L)	49.5 ± 0.29^a^	34.6 ± 0.37^c^	39.7 ± 0.35^ab^	30.3 ± 0.27^d^
AST (U/L)	282.6 ± 0.35^a^	233.4 ± 0.04^c^	244.3 ± 7.18^b^	229 ± 0.57^c^
Bilirubin total (mg/dL)	0.067 ± 0.002^a^	0.054 ± 0.003^b^	0.06 ± 0.004^a^	0.019 ± 0.03^d^
Alkaline phosphate (U/L)	299 ± 4.03^a^	216 ± 4.99^c^	292 ± 3.10^a^	283 ± 3.36^b^
Total serum protein (g/dL)	6.94 ± 0.02^ab^	6.94 ± 0.03^a^	6.75 ± 0.03^c^	7.04 ± 0.03^a^
**Renal function (mg/dL)**				
Creatinine	0.36 ± 0.02^c^	0.47 ± 0.04^a^	0.39 ± 0.04^b^	0.37 ± 0.03^c^
BUN	18.65 ± 0.20^a^	17.17 ± 0.45^b^	17.23 ± 0.90^b^	17.08 ± 0.57^c^
Uric acid	0.92 ± 0.04^ab^	0.72 ± 0.04^d^	0.81 ± 0.03^c^	1.20 ± 0.31^a^
**Lipid Profile (mg/dL)**				
Cholesterol	111.00 ± 5.29^a^	79.65 ± 0.20^b^	77.12 ± 0.85^b^	67.27 ± 0.42^d^
HDL	45.08 ± 0.14^c^	52.27 ± 0.27^a^	48.40 ± 0.36^ab^	47.10 ± 0.57^b^
LDL	32.30 ± 0.31^a^	19.50 ± 0.25^c^	27.75 ± 0.42^b^	15.45 ± 0.42^d^
Triglyceride	42.65 ± 0.47^a^	44.47 ± 0.29^a^	41.27 ± 0.35^b^	38.27 ± 0.40^c^

*Note:* NC, Normal control with 0 g of Coriander leaf powder, Coriander Treatment 1 (CT_1_) with 12.2 g/100 g coriander leaf powder, Coriander Treatment 2 (CT_2_) with 16.26 g/100 g coriander leaf powder, and Coriander Treatment 3 (CT_3_) with 20.33 g/100 g coriander leaves powder. Data was analyzed by using one‐way ANOVA, followed by multiple comparisons using LSD, and data was considered significant at *p* ≤ 0.05*. *p* ≤ 0.05 means significant, and *p* ≥ 0.05 means results are non‐significant. Mean within the same column with different superscripts (a‐d) is statistically different at *p* ≤ 0.05.

Abbreviations: ALT, aminotransferase; AST, aspartate aminotransferase; BUN, blood urea nitrogen; HDL, high‐density lipid; LDL, low‐density lipid.

### 3.7 Effect of Coriander on Serum Uric Acid and Renal Function

Results regarding the renal parameters showed an ameliorative effect among the treatment groups (*p* ≤ 0.05). There was a significant decrease (17.08 ± 0.57 mg/dL), (0.72 ± 0.04 mg/dL) (*p* ≤ 0.05) in BUN. The uric acid level significantly decreased in rats fed the CT_1_ (0.72 ± 0.04 mg/dL) and CT_2_ (0.81 ± 0.03 mg/dL) diets. However, no change was observed in the CT_3_ group (1.20 ± 0.31 mg/dL) compared to the NC group (0.92 ± 0.04 mg/dL). Creatinine levels were found non‐significant in all treatment groups because there was no effect on these values (*p* ≥ 0.05) (Table 6).

### 3.8 Effect of Coriander on Lipid Profile

Similarly, lipid levels were also attenuated in the treatment groups. Serum cholesterol significantly decreased (*p* ≤ 0.05) in rats offered coriander diets CT_3_ (67.27 ± 0.42 mg/dL) and CT_2_ (77.12 ± 0.85 mg/dL). A similar decreasing trend was seen in values of LDL and triglycerides and a higher ameliorative effect (*p* ≤ 0.05) of both (15.45 ± 0.42 mg/dL), (38.27 ± 0.40 mg/dL) was observed in rats offered the CT_3_ diet. However, HDL level was improved (*p* ≤ 0.05) in the treatment groups, and an improved effect was seen (52.27 ± 0.27 mg/dL), (48.40 ± 0.36 mg/dL) in CT_1_ and CT_2_ groups. (Table 6).

### 3.9 Effect of Different Levels of Coriander on Body Immunity and Hematology

White blood cell count significantly improved (*p* ≤ 0.05) (14.63 ± 0.07 × 10^3^ μL), (7.30 ± 0.25 × 10^3^ μL) in rats fed CT_3_ and CT_1_ diets when compared to the NC (8.68 ± 0.07 × 10^3^ μL), whereas CT_2_ (6.92 ± 0.04 × 10^3^ μL) diets did not show an increasing trend according to Table 7. Neutrophils were significantly found higher (59.81% ± 0.10%) and (42.02% ± 0.15%) in rats fed CT_2_ and CT_1_ diets in comparison with the CT_3_ (34.11% ± 0.07%) and NC (40.60% ± 0.07%). Lymphocyte percentage was found non‐significant (57.80% ± 0.13%), (49.29% ± 0.08%), (53.55% ± 0.65%) in rats fed CT_3_, CT_1_, and NC diets, but CT_2_ (33.88% ± 0.09%) had shown decreasing trend. Similarly, a significantly increasing trend was observed in monocyte percentage among the coriander‐treated levels. Red blood cell count was significantly increased in coriander‐treated diets, but significant increases (8.63 ± 0.05 × 10^6^ μL), (7.94 ± 0.02 × 10^6^ μL) in RBCs were observed in rat‐fed CT_2_ and CT_1_ diets in comparison with the CT_3_ (7.05 ± 0.11 × 10^6^ μL) and NC (6.51 ± 0.10 × 10^6^ μL). In a similar context, coriander‐treated diets had a favorable influence on Hb levels, and significantly higher (15.04 ± 0.13 g/dL) and (13.20 ± 0.43 g/dL) values were found in rats fed CT_2_ and CT_1_ diets when compared to the NC (11.72 ± 0.12 g/dL). In terms of platelet count, improved results (865.00 ± 5.29 × 10^5^/μL), (672.50 ± 4.65 × 10^5^ μL) were observed in rats offered CT_3_ and CT_2_ diets in comparison with the CT_1_ (262.00 ± 5.16 × 10^5^/μL) and NC (262.00 ± 5.16 × 10^5^/μL) diets.

**TABLE 7:** Effect of coriander on body immunity and hematology biomarkers.

Parameters	Groups			
	NC	CT_1_	CT_2_	CT_3_
White blood count (×10^3^ μL)	8.68 ± 0.07^b^	7.30 ± 0.25^b^	6.92 ± 0.04^c^	14.63 ± 0.07^a^
Neutrophils %	40.60 ± 0.07^b^	42.02 ± 0.15^b^	59.81 ± 0.10^a^	34.11 ± 0.07^d^
Lymphocytes %	53.55 ± 0.65^a^	49.29 ± 0.08^b^	33.88 ± 0.09^c^	57.80 ± 0.13^a^
Monocytes %	2.10 ± 0.06^c^	4.70 ± 0.08^a^	2.41 ± 0.10 ^b^	2.11 ± 0.09^c^
RBC (×10^6^ μL)	6.51 ± 0.10^c^	7.94 ± 0.02^b^	8.63 ± 0.05^a^	7.05 ± 0.11^b^
Hb (g/dL)	11.72 ± 0.12^d^	13.20 ± 0.43^b^	15.04 ± 0.13^a^	12.42 ± 0.40^b^
Platelet (×10^5^ /μL)	512.75 ± 2.98^c^	262.00 ± 5.16^d^	672.50 ± 4.65^b^	865.00 ± 5.29^a^

*Note:* NC, Normal control with 0 g Coriander leaf powder, Coriander Treatment 1 (CT_1_) with 12.2 g/100 g coriander leaf powder, Coriander Treatment 2 (CT_2_) with 16.26 g/100 g coriander leaf powder and Coriander Treatment 3 (CT_3_) with 20.33 g/100 g coriander leaves powder. Data was analyzed by using one‐way ANOVA, followed by multiple comparisons using LSD, and data was considered significant at *p* ≤ 0.05*. *p* ≤ 0.05 means significant, and *p* ≥ 0.05 means results are non‐significant. Mean within the same column with different superscripts (a–d) is statistically different at *p* ≤ 0.05.

Abbreviations: Hb, hemoglobin; RBC, red blood cells.

## 4 Discussion

Coriander leaf powder significantly reduces uric acid in healthy Wistar albino rats, improving other parameters and indicating acceptance of the hypothesis. This study found a positive effect of coriander on feed intake, body weight, nutrient intake, and digestibility. Specifically, we were concerned with the various levels of coriander that contain quercetin and its therapeutic effect. Previous literature mostly focused on in vitro studies, while this study is advanced because we conducted an in vivo trial. This study offers coriander with various levels of quercetin, which is important to determine the best level for healthy individuals to prevent disease. We conducted the study on healthy albino rats to determine the best level of treatment compound for the prevention of hyperuricemia because the level of phenolic compounds in various herbs and foods is different for healthy and diseased conditions. The study provides a solution for the prevention of hyperuricemia by determining the optimal level of quercetin in coriander that can be added to the daily diet to prevent the occurrence of hyperuricemia. However, the study had some limitations, particularly since it was conducted in the context of “prevention is better than treatment,” and hyperuricemia was not induced in the animals. This study also demonstrated the approximate composition of coriander leaf with a moisture content of (86.96%), CP (15.31%), EE (0.89%), CF (6.02%), ash (1.8%), and NFE (carbohydrates) of (10.98%) in comparison to Shahwar et al. (2012), who evaluated the nutritional value of fresh coriander leaves. These findings are consistent with their study, which found that coriander leaf had more nutritional content than our study did in terms of CP (4.05%), CF (5.24%), NFE (1.15%), moisture (86.71%), EE (0.95%), and ash (1.9%). Supporting our results, Mouhoubi et al. (2023) claimed a positive impact on the phenolic content of coriander leaves, which was characterized by using UHPLC. This study might be connected to that of Hashim et al. (2005) who found quercetin levels in coriander stem and leaf varied from 49.8 to 397.5 mg/100 g of dry extract. Further research by Pandey et al. (2011), Barros et al. (2012), and Tang et al. (2013) demonstrated the presence of isoquercetin and quercetin in an ethanolic extract of coriander leaves by HPLC fingerprinting. The coriander plant's vegetative parts (leaves and stems) contained 3296 mg/kg dw of quercetin‐3‐O‐rutinoside, among other phenolic substances, flavonoids, and quercetin derivatives. Various plants and vegetables were also shown to contain quercetin (David et al. 2016; Batiha et al. 2020). The results of the HPLC analysis of the coriander leaf powder extract on a dry matter basis are shown in Figure 2A,B. The results revealed a significant peak with a peak area of 135,267.5 and height 9372.2 and a retention time *t*_*R*_ of 16.371 at 360 nm. As evidenced by their peak areas and heights of 143,558.6 and 12,760.1 in comparison to the quercetin standard and a retention time *t*_*R*_ of 16.954 at 360 nm. Therefore, based on HPLC analysis, this study found that coriander leaves contained 18.82 mg of quercetin per 100 g dry weight. These study results align with Hussain, Arif, et al. (2024) and Hussain, Korma, et al. (2024), who investigated the drying methods of microwave and ultrasonic‐assisted techniques on coriander leaves, flowers, and seeds to identify various functional compounds. They found a positive effect on the nutritional value of coriander, along with an increase in total phenolic content, total flavonoid content, and overall antioxidant activity. This is non‐significant with this study which adopted the oven drying method, also revealed a positive impact on the nutritional value of coriander leaves. After quantification via HPLC‐UV, it showed enormous levels of quercetin and other phenolic compounds. Similar to this, Verma and Trehan (2013) used HPLC to discover quercetin concentration in the methanolic extract of coriander. HPLC results also declare the amount of quercetin compounds was 6.10 mg/kg, with a peak area and retention time of 8,796,752 and 17.249, respectively. In contrast to earlier research, the variation in coriander quantity may depend on changes in coriander variety, regional, and environmental factors. The main sources of quercetin are onions, apples, and berries. Coriander leaves also contain varying amounts of quercetin, which has shown promising effects in treating a range of ailments (Shoko et al. 2022; Aghababaei and Hadidi 2023; Nambiar, Daniel, and Guin 2010).

Up to the second week, feed intake was non‐significant (*p* ≥ 0.05), and in the last week, there was rarely an increase. During treatment, BW was significantly reduced, and FCR and FER were also potentially affected. Furthermore, Hernandez et al. (2004) hypothesized that three factors might be related to a drop in BW. (1) The greater fiber content of coriander and feed results in a satiety effect and reduces BW by influencing appetite (El‐Kherbawy, Ibrahem, and Zaki 2011). (2) The coriander's quercetin and flavonoids have anti‐lipidemic effects, which lower lipid uptake and enhance liver fat metabolism (Moharib and Adly 2024; Dhanapakiam et al. 2008; Wang et al. 2019). (3) Because more nitrogen was excreted than was consumed, a negative nitrogen balance may also be the reason for a decrease in BW (Tebib et al. 1994; Đurendić‐Brenesel et al. 2013). According to study results by Kamel (2000), herbal mixtures with antibacterial and antifungal activities may have had a good impact on growth efficiency and digestibility. Significant reductions in BW and FER were observed in the rats fed the tested compounds, which may be related to the higher fiber content in the coriander and parsley‐supplemented diet Liu et al. (2013). Because coriander improves the digestion of protein and fiber, water consumption was also increased. It may occur because a diet rich in protein and fiber needs more water for the digestive enzymes to function. Common carp ( *Cyprinus carpio* ) ate their food with greater interest when quercetin was given to them at doses of 200 and 800 mg/kg (Ghafarifarsani et al. 2022). (Table 2) In the last week of the trial, after the collection period, the nutrient intake (g) among the treatment groups was found to be optimum, with the highest intake observed in rats fed the CT_3_ diet, followed by the CT_1_ group. Similar results were seen in the case of NFE intake, but their values are non‐significant in NC and CT_2_ (Table 3) (Umer et al. 2023, 2024). Nutrient intake can be enhanced by various factors such as improved feed intake and digestibility, enhanced digestive enzymes, and the beneficial effect on gut peristalsis movement due to the high fiber content in coriander leaves. Although digestibility was found to be lower than in rats fed the CT_1_ diet, DM intake increased substantially in rats fed the CT_3_ diet. This could be because the coriander‐treated meals contained more fiber. Fiber's satiety effect resulted in a decrease in DM intake. The liver may have performed better because the coriander‐treated diets significantly improved protein intake and digestibility. For several metabolic processes, such as maintaining and repairing human tissues, the body can more easily catalyze the conversion of protein into simple amino acids when it is more easily digestible. The liver contributes significantly to the regulation of protein metabolism by synthesizing serum total protein (albumin) and enhancing the activity of several enzymes. The action of amylase enzymes accelerated the digestion of NFE (carbohydrates) (Umer et al. 2023, 2024). Coriander‐treated meals showed higher fiber intake and digestibility, which increases the gut microbes activity and encourages them to produce volatile fatty acids in the colon after fiber gets broken down. Quercetin functions as a prebiotic in diets that have been treated with coriander to make fiber easier to digest by encouraging the growth and activity of beneficial microbes (Liu et al. 2014). Phytogenic compounds are now recognized as a beneficial feed component for the chicken industry due to their capacity to promote hunger, scavenge free radicals, and function as an antibacterial agent (Akhtar et al. 2023; Mountzouris et al. 2007). Another study discovered that coriander has antifungal, antibacterial, and antilipidemic traits, making it a useful food for improving appetite and digestion (Delaquis et al. 2002). Quercetin is a flavanol component that promotes health by modulating the function of digestive enzymes while encouraging the development of healthy bacteria in the colon. It also enhanced stomach absorption by expanding the villi (Brush borders) and giving more surface area for higher absorption (Khalil et al. 2020; Liu et al. 2011). As was previously determined, quercetin affects the number and activity of beneficial microbes (Lactobacilli) while harming pathogenic microbes like *C. perfringens* (coliform count) (Liu et al. 2014). Similar to this, 200 mg of quercetin per bird markedly increased crypt depth and villus height, maintained better digestive health in birds, and improved absorption status. By expressing the genes for nutrient transporters like GLUT2, PEPT1, and FAS, quercetin also improved nutrient absorption and digestion (Teirlynck et al. 2009; Sukhotnik et al. 2018). When given to broiler chicks at doses of 0.25, 0.50, or 100 g/kg, quercetin of plant origin exhibits improved effects on growth performance, cecal microbiota status, and organ weight (Zhang and Kim 2020). By causing the production of additional bile acids and pancreatic enzymes, coriander facilitates the absorption and digestion of lipids (Lee 2002) (Table 4).

In comparison with the other treatment diets, rats offered the CT_2_ diet revealed positive nitrogen balance, but rats fed the CT_1_ and CT_2_ diets showed negative nitrogen balance (Table 5). Digestibility and protein intake appear to be better at these levels; however, as is well known, serum urea depends on the type and amount of protein consumed. Rats fed diets treated with coriander experienced both positive and negative nitrogen balances for several reasons. Because coriander contains antinutritional substances such as phytates, oxalate, and coumarins that bind to proteins and reduce their availability for absorption and metabolism, it may have a negative nitrogen balance. When a person loses weight, the body's ability to absorb protein is also reduced while fewer calories are consumed, which causes a negative nitrogen balance (Kaneko 1989; Iyayi and Tewe 1998). The phenolic components and flavonoids found in coriander, such as quercetin, kaemferol, rhamnetin, and apigenin, which have free radical scavenging and antioxidant activities, have already been discussed (Nworgu, Ogungbenro, and Solesi 2007; Rajeshwari and Andallu 2011). Additionally, it decreased BUN and renal indices; however in this trial, creatinine and BUN levels were much greater than those in NC. When discussing nitrogen balance, both BUN and creatinine play different roles. A spike in BUN is a sign that you're eating a lot of protein, which means your liver is metabolizing more of it, which leads to a positive nitrogen balance. The end product of creatinine phosphate from muscle tissue is creatinine; if renal insufficiency develops, nitrogenous end products from metabolism may not be able to be eliminated from the body, leading to a positive nitrogen balance. Nisa et al. (2006) research indicates that the slower release of nitrogen linked to fiber corresponded with fiber fermentation and its consumption by ruminal microorganisms, limiting loss. Similar to this, the fiber in coriander and feed may provide the gut microorganisms more power so they can hold onto more protein after the process of gluconeogenesis results in the production of volatile fatty acids in the colon following the digestion of fiber. Short‐chain fatty acids (SCFAs), which are produced by fiber in the gut and provide animals energy, are made possible by the fermentation activities of the gut microbiota. Fiber also strengthens the intestinal lining for improved digestion and absorption. The results of our research corroborate the findings of Metzler, Bauer, and Mosenthin (2005), who offered phytogenic herbal supplements to hens at doses of 0.5, 1.0, and 1.5 kg/ton. By enhancing the activity of digestive enzymes, which play a role in protein digestion and absorption, herbal supplements help the body retain more nitrogen in the gut and make it easier to digest proteins. This may be related to the herbal supplement's antimicrobial properties. Cho et al. (2017) provided Korean indigenous goats with quercetin at varied concentrations (0, 500, and 1000 ppm) by varying the amount of concentrate and roughage (RC ratio). Due to their antioxidant and anti‐inflammatory activities, the addition of quercetin at a level of 500 ppm has demonstrated a favorable nitrogen balance.

As indicated in Table 6, coriander has antioxidant properties and may improve hepatic morphology by decreasing bilirubin and liver enzyme levels. It also has an antilipidemic action, assisting in the condition of fatty liver. There are many different medicinal and therapeutic substances in coriander, such as phospholipids, phytosterols, and flavonoids with anti‐inflammatory and antioxidant properties (Akhtar et al. 2023; Henry, Neil, and William 2003; Zengin et al. 2011). In the present investigation, rats fed diets treated with coriander had a beneficial effect on hepatic enzyme levels; however, certain values are non‐significant when compared to the NC diet. The levels of bilirubin in two groups of rats fed the diets CT_2_ and CT_3_ did not change. The anti‐nutritional coriander ingredients phytates and oxalate, which restrict quercetin's availability for its therapeutic activity, may be the cause of this variance. Because there are more RBCs available for the endothelium system to use in the production of bilirubin, an increase in RBCs could be responsible for an increase in bilirubin in various treatment levels. Another possibility is that the CT_2_ and CT_3_ meals included higher coriander levels, which may have altered the liver and gallbladder functioning. Coriander and quercetin improve the endothelium system and the outcome of bilirubin metabolism as a result of their antioxidant action (Kumar Mishra, Singh, and Rath 2013). In the observation of Fahmy, Shreif, and Gharib (2014), rats who received toxicity‐induced radiation showed improved effects on serum liver enzymes and serum total protein when given coriander leaf extract 600 mg/kg and silymarin 70 mg/kg daily for 10 days. In an accompanying inquiry, rats given CCl‐induced toxicity received intraperitoneal injections of coriander extract at doses of 100, 200, and 300 mg/kg. The effect of 300 mg of coriander extract on serum total protein, ALT, AST, ALP, and direct and indirect bilirubin was found positive. While coriander's quercetin concentration was quantified in the same study, it was found to range from 40.8 to 397.5 mg/100 g of dried extract (Pandey et al. 2011). Rats treated with 200 mg/kg of thioacetamide (TAA) per kilogram were fed coriander twice a week, and the liver parameters improved (Moustafa et al. 2014). In another trial, Japanese quail were fed coriander leaf meal for eight weeks at levels of 0%, 2%, 4%, and 6%. Quails that were fed a diet containing 2% coriander meal had significantly lower serum levels of ALT, AST, ALP, total protein, and bilirubin (Alagbe 2018). Due to the recommended dietary allowance of protein offered in the meal, serum protein levels also improved (Umer et al. 2023). Zhang et al. (2006) added quercetin's defense against the toxicity of iron excess on hepatic indices in mice. Lipid peroxidation is a series of chain reactions that causes oxidative damage to the phospholipid cell membrane in the liver. Another study supports our findings, in which hyperlipidemic rats were provided with a coriander‐enriched diet containing different levels of coriander powder and extract. A more promising effect was observed on the levels of liver enzymes ALT and AST. Rats that were given coriander powder in their diet showed a significant impact compared to those given coriander extract. Coriander is a good source of polyphenols and phytochemicals such as quercetin, flavonoids, coumarins, and cinnamic acid, which have antioxidant properties that are beneficial for the cellular texture and morphology of liver cells (Anfenan 2014). The antioxidant effects of quercetin and coriander, which protect from free radical exposure and enhance the liver's capacity to produce more protein, may be liable for the rise in blood total protein. This study shows rats fed a coriander diet had a significant decrease in uric acid, however, coriander at different doses had no effect on BUN or creatinine. When there was a negative nitrogen balance, the body excreted more nitrogen than it takes in. As noted in Table 6, despite the body excreting more nitrogen, BUN and creatinine levels were still higher in this study. This occurs because the body's breakdown of protein results in an increase in BUN and creatinine. The body obtains its energy needs to manage the condition from the breakdown of the muscle tissues, which causes an increase in creatinine levels. The other reason is because of increased protein breakdown, which puts too much strain on the kidneys to eliminate the end products of protein, leading to an increase in renal parameters (Kenelly et al. 2018). Rats fed the CT_1_ and CT_2_ diets had significantly lower uric acid levels than those fed the CT_3_ and NC diets. The fact that the uric acid level did not decrease with the CT_3_ diet may be related to quercetin's lesser affinity for the xanthine oxidase enzyme as a result of the presence of coriander anti‐quality ingredients such oxalate, phytate, and alkaloids that limited the free availability of nutrients and quercetin. Another acceptable explanation is that the body's negative nitrogen balance leads more nitrogen to be excreted and less protein is available for protein breakdown, which lowers uric acid levels. Rats fed 7.0 g/kg parsley showed a significantly more reduction in serum uric acid levels followed by a reduction in the level of 3.5 g/kg parsley (Rahmat, Ahmad, and Ramli 2019). Similar to this study, Soliman et al. (2020) stated that parsley and celery offered at 7 g/kg BW and 500 mg/kg orally for 10 days had a positive impact on BUN, serum uric acid, and hepatic xanthine oxidase enzyme. Our findings are supported by another study that demonstrates quercetin decreases free radical generation in kidney damage brought on by gentamicin GM. Additionally, it reduces levels of creatinine and BUN and prevents the oxidation of lipids and xanthine oxidase (Endrini et al. 2023; García‐Nieto 2022; Abdel‐Raheem, Abdel‐Ghany, and Mohamed 2009).

Hyperlipidemia is linked to elevated levels of cholesterol and triglycerides. LDL is considered a bad lipoprotein, as it promotes atherosclerosis by forming plaque in the arteries, leading to an increased risk of cardiovascular events (Frankel et al. 1993). In contrast, HDL is a good lipoprotein and has a positive impact on atherosclerosis because it removes lipid deposits from the arteries. In our study, various levels of coriander had a positive effect on serum lipid levels. This might be due to coriander's capacity to increase lipid catabolism by enhancing their metabolism and excretion from the liver (Sorial 2021; Anfenan 2014). Due to improved intake of fiber and its digestibility, which binds lipid molecules in the intestine and decreases lipid levels in the body, coriander‐treated diets showed better blood cholesterol and LDL levels. By regulating inflammation and multiple metabolic processes in the body, beneficial microbes in the gut additionally impact how well dietary fiber works in the body. Along with all of this, the presence of chlorophyll, phytosterol, and fiber content in coriander, which showed their presence at 6.02% (fiber) in this study, also played a supporting function (Glore et al. 1994). LDL and very low‐density lipoprotein (VLDL) cholesterol levels drop as a result of coriander's increased fecal production of bile acid and cholesterol (Gordon 1992). Additionally, it lessens the activity of HMG‐CoA reductase in the liver, a coenzyme that is vital to the production of lipids (Lee et al. 2008). Previous studies showed that quercetin decreased cholesterol levels by reducing de novo lipid synthesis and activity of the lipase enzymes acetyl‐CoA carboxylase (ACC) and diacylglycerol acyltransferase (DGAT) (Qureshi et al. 2011; Gnoni, Paglialonga, and Siculella 2009), as mentioned in Table 6. The LDL and triglyceride levels were high in CT_2_ and CT_1_ might be related to the low bioavailability of phenolic compounds in the body, which can form complexes with other substances and limit the availability of phenolic and flavonoid compounds for metabolism (Natesh, Abbey, and Asiedu 2017; Aguerre et al. 2016). Other factors that could contribute to disturbances in lipid metabolism, coriander‐to‐feed ratio, the temperament of the rats, and the nutrient composition of their feed (Gruendel et al. 2006; Hwang et al. 2001). This might also be associated with the disturbance in the gut microbiota flora, which deals with the fermentation of fiber content in the coriander and feed. Various studies have explored different formulations, such as nanoparticles and emulsions, to enhance the bioavailability of phenolic compounds.

By synthesizing antibodies, storing platelets, homeostasis of bilirubin, producing albumin and globulin, detoxifying medicines and poisons, and storing vitamins and minerals, the liver and total protein in the diet play a crucial role in maintaining hematological traits. As shown in Table 7, the addition of coriander to the diet had a substantial impact on these values in this study. The hematological characteristics RBCs, WBCs, and platelet count have been investigated as significant risk factors in a variety of clinical diseases (Farag et al. 2021). Due to their unique morphology, RBCs are crucial in the delivery of nutrients and oxygen throughout the body. They are more vulnerable to exposure to free radicals that damage their morphology; due to their antioxidant activity, coriander protects the integrity of RBCs (Barbalato and Pillarisetty 2020). This study would be linked with Ufele, Mgbenka, and Ude (2008), who found that mice given different amounts of plant‐based proteins such as soybean and maize meal had significantly higher (*p* ≤ 0.05) RBC levels. Plant protein has a bioavailability of 70% compared to animal protein's 86%; yet, in this trial, a meal high in plant protein had a favorable impact on RBC levels. WBCs were significantly increased in rats fed the CT_3_ diet, whereas other treatment diets had shown negligible improvement in WBCs but were found nonsignificant (*p* ≥ 0.05) to the NC diet. The coriander's anti‐inflammatory and antioxidant properties may be the reason for this improvement. As already pointed out, when compared to the NC diet, the WBCs from the CT_1_ and CT_2_ diets were found to be non‐significant (*p* ≥ 0.05); this might be due to antinutritious substances such as phytates, oxalates, and coumarins in coriander that bind the important functional compounds like quercetin. The coriander diet's higher coumarin level could cause a thinning effect on the blood. These binding substances in this investigation might be the reason why the hematological levels remained constant when compared to the NC (Alagbe 2018). Adeyinka and Bello (2013) noted that WBCs and other leukocytes can combat various infections and produce antibodies to defend the body. The essential role of hemoglobin in animals is to carry oxygen to the cells. It is a key marker for nutritional assessment in the body. Neutrophil and lymphocyte percentages that rise are good indications of reduced nutritional stress, but quercetin and coriander's therapeutic effects also help these cells function more efficiently in the body (Adekoya et al. 2009). This study's results contradict Rafiu et al. (2013) and Olufemi et al. (2003), who revealed no beneficial effects on hematological traits when Amaranthus spinosus leaf powder was incorporated into the diet of developing pigs. Our results follow the findings of Selvakumar et al. (2013), who found quercetin improved the hematological values in Wistar albino rats. It reduces inflammation and strengthens the immune system; additionally, it also raises WBCs and lymphocyte percentages (Karuppasamy, Subathra, and Puvaneswari 2005; Donmez et al. 2019) and (Fahim et al. 2012).

## 5 Conclusion

The results indicated that CT_1_ and CT_2_ diets, which included coriander leaf powder at concentrations of 12.2 g/100 g of feed and 16.26 g/100 g of feed, corresponding to doses of quercetin at 0.075 and 0.100 mg/kg, had a positive impact on serum uric acid levels, serum, and hematological parameters. There was a favorable effect on weight management parameters, nutrient intake, digestibility, and modulation of nitrogen metabolism. The studies signify that coriander leaf powder contains quercetin at 18.82 mg/100 g on a dry matter basis, quantified via HPLC‐UV. Therefore, coriander, an important culinary herb, can prevent and cure a wide range of illnesses; it can also be used for the occurrence and treatment of hyperuricemia and other related disorders.

### 5.1 Recommendations

To validate the efficacy and therapeutic effect of coriander leaf, experiments should be performed on a specific group of humans by inducing uric acid with dietary modifications. After inducing hyperuricemia in the long term, the dose of coriander should be offered on an edible or dry matter basis. From our study trial results, we have calculated the best human equivalent dose of coriander leaf on an “as such” and dry matter basis corresponding to the levels of quercetin present in it. This dose might be good for the prevention of hyperuricemia if it is taken with the meal. It would be best to examine the XO inhibitory activity of coriander leaf powder and quercetin in vitro and observe the morphological and structural relationship with the XO via molecular docking.

## Author Contributions

**Mahr‐Un Nisa:** conceptualization (lead), investigation (equal), methodology (equal), resources (equal), supervision (lead), visualization (equal), writing – review and editing (equal). **Muhammad Umer:** conceptualization (equal), data curation (lead), formal analysis (lead), investigation (lead), methodology (equal), resources (equal), writing – original draft (equal), writing – review and editing (equal). **Muhammad Hamza:** formal analysis (equal), investigation (equal), validation (equal), visualization (equal), writing – review and editing (equal). **Huma Umbreen:** formal analysis (equal), resources (equal), validation (equal), visualization (equal), writing – review and editing (equal). **Nukhba Khalid:** formal analysis (equal), methodology (equal), resources (equal), writing – review and editing (equal). **Muhammad Qasim Raza:** data curation (equal), formal analysis (equal), resources (equal), writing – review and editing (equal). **Isam A. Mohamed Ahmed:** formal analysis (equal), methodology (equal), resources (equal), validation (equal), visualization (equal), writing – review and editing (equal). **Moneera O. Aljobair:** formal analysis (equal), funding acquisition (equal), resources (equal), validation (equal), visualization (equal), writing – review and editing (equal). **Osman Ahmad Khan:** formal analysis (equal), resources (equal), visualization (equal), writing – review and editing (equal).

## Ethics Statement

The experimental procedure was approved by the Animal Ethical Committee of Government College University, Faisalabad, Punjab, Pakistan.

## Conflicts of Interest

The authors declare no conflicts of interest.

## Data Availability

The supporting data of the findings of the present study are available from the corresponding author upon reasonable request.

## References

[fsn370029-bib-0001] David, A. V. A. , R. Arulmoli , and S. Parasuraman . 2016. “Overviews of Biological Importance of Quercetin: A Bioactive Flavonoid.” Pharmacognosy Reviews 10, no. 20: 84. 10.4103/0973-7847.194044. 28082789 PMC5214562

[fsn370029-bib-0002] Abdel‐Latif, M. A. , A. R. Elbestawy , A. H. El‐Far , et al. 2021. “Quercetin Dietary Supplementation Advances Growth Performance, Gut Microbiota, and Intestinal mrna Expression Genes in Broiler Chickens.” Animals 11, no. 8: 2302. 10.3390/ani11082302. 34438756 PMC8388376

[fsn370029-bib-0003] Abdel‐Raheem, I. T. , A. A. Abdel‐Ghany , and G. A. Mohamed . 2009. “Protective Effect of Quercetin Against Gentamicin‐Induced Nephrotoxicity in Rats.” Biological and Pharmaceutical Bulletin 32, no. 1: 61–67. 10.1248/bpb.32.61. 19122282

[fsn370029-bib-0115] Adekoya, A. Y. , A. Y. Ayo , A. K. B. Sackey , and A. B. Adelaiye . 2009. “Haematological Changes in Pigs Administered with Ascorbic Acid and Transported by Road for Four Hours During the Harmattan Season.” Journal of Cell and Animal Biology 3, no. 2: 21–28.

[fsn370029-bib-0005] Adeyinka, J. N. , and H. O. Bello . 2013. “Serum Biochemical Parameters in Clinically Healthy Dogs in Ibadan.” Tropical Veterinarian 16: 123–129.

[fsn370029-bib-0006] Aghababaei, F. , and M. Hadidi . 2023. “Recent Advances in Potential Health Benefits of Quercetin.” Pharmaceuticals 16, no. 7: 1020. 37513932 10.3390/ph16071020 PMC10384403

[fsn370029-bib-0007] Aguerre, M. J. , M. C. Capozzolo , P. Lencioni , C. Cabral , and M. A. Wattiaux . 2016. “Effect of Quebracho‐Chestnut Tannin Extracts at 2 Dietary Crude Protein Levels on Performance, Rumen Fermentation, and Nitrogen Partitioning in Dairy Cows.” Journal of Dairy Science 99, no. 6: 4476–4486. 27060814 10.3168/jds.2015-10745

[fsn370029-bib-0008] Akhtar, M. S. , M. Rafiullah , M. A. Hossain , and M. Ali . 2023. “Antidiabetic Activity of *Cichorium intybus* L Water Extract Against Streptozotocin‐Induced Diabetic Rats.” Journal of Umm Al‐Qura University for Applied Sciences 9, no. 4: 565–571. 10.1007/s43994-023-00066-1.

[fsn370029-bib-0009] Alagbe, J. O. 2018. “Effect of Different Levels of Feed Added Coriander ( *Coriandrum sativum* ) Leaves Meal on the Performance, Carcass Quality, Immune Response and Blood Profile of Quails (*Corturnix cortunix japonica*).” Pacific International Journal 1, no. 4: 142–150. 10.55014/pij.v1i4.46.

[fsn370029-bib-0010] Anfenan, M. L. K. 2014. “Study the Effect of Consumption of Coriander and Vitamin B6 on Rats Suffering From Hyperlipidemia.” World Applied Sciences Journal 30: 1504–1509.

[fsn370029-bib-0011] AOAC . 2006. Official Methods of Analysis. Association of Official Analytical Chemists.

[fsn370029-bib-0012] Aoun, R. , F. A. Z. Chokor , M. Taktouk , et al. 2022. “Dietary Fructose and Its Association With the Metabolic Syndrome in Lebanese Healthy Adults: A Cross‐Sectional Study.” Diabetology & Metabolic Syndrome 14, no. 1: 29. 10.1186/s13098-022-00800-5. 35139893 PMC8827166

[fsn370029-bib-0013] Barbalato, L. , and L. S. Pillarisetty . 2020. “Histology, Red Blood Cell.” In StatPearls. StatPearls Publishing. 30969524

[fsn370029-bib-0014] Barros, L. , M. Duenas , M. I. Dias , M. J. Sousa , C. Santos‐Buelga , and I. C. Ferreira . 2012. “Phenolic Profiles of In Vivo and In Vitro Grown *Coriandrum sativum* L.” Food Chemistry 132, no. 2: 841–848. 10.1016/j.foodchem.2011.11.048.

[fsn370029-bib-0015] Batiha, G. E. S. , A. M. Beshbishy , M. Ikram , et al. 2020. “The Pharmacological Activity, Biochemical Properties, and Pharmacokinetics of the Major Natural Polyphenolic Flavonoid: Quercetin.” Food 9, no. 3: 374. 10.3390/foods9030374. PMC7143931 32210182

[fsn370029-bib-0016] Bhat, S. , P. Kaushal , M. Kaur , and H. K. Sharma . 2014. “Coriander (*Coriandrum sativum* L.): Processing, Nutritional and Functional Aspects.” African Journal of Plant Science 8, no. 1: 25–33. 10.5897/AJPS2013.1118.

[fsn370029-bib-0018] Caliceti, C. , D. Calabria , A. Roda , and A. F. Cicero . 2017. “Fructose Intake, Serum Uric Acid, and Cardiometabolic Disorders: A Critical Review.” Nutrients 9, no. 4: 395. 10.3390/nu9040395. 28420204 PMC5409734

[fsn370029-bib-0019] Chhapra, I. U. , A. Mashkoor , and N. A. Syed . 2010. “Changing Sugar Consumption Pattern in Pakistan and Increasing Sugar Industry's Profitability.” IBT Journal of Business Studies 2, no. 2: 52–64. 10.46745/ilma.jbs.2010.06.02.01.

[fsn370029-bib-0020] Cho, C. H. , B. M. Yang , N. S. Park , et al. 2017. “Relationship Linking Dietary Quercetin and Roughage to Concentrate Ratio in Feed Utilization, Ruminal Fermentation Traits and Immune Responses in Korean Indigenous Goats.” Journal of the Korean Society of Grassland and Forage Science 37, no. 1: 10–18. 10.5333/KGFS.2017.37.1.10.

[fsn370029-bib-0021] Delaquis, P. J. , K. Stanich , B. Girard , and G. Mazza . 2002. “Antimicrobial Activity of Individual and Mixed Fractions of Dill, Cilantro, Coriander and Eucalyptus Essential Oils.” International Journal of Food Microbiology 74, no. 1–2: 101–109. 10.1016/s0168-1605(01)00734-6. 11929164

[fsn370029-bib-0116] Dhanapakiam, P. , J. M. Joseph , V. K. Ramaswamy , M. Moorthi , and A. S. Kumar . 2008. “The Cholesterol Lowering Property of Coriander Seeds (*Coriandrum sativum*): Mechanism of Action.” Journal of Environmental Biology 29, no. 1: 53–56. 18831331

[fsn370029-bib-0023] Donmez, H. H. , N. Donmez , I. Kısadere , and I. Undag . 2019. “Protective Effect of Quercetin on Some Hematological Parameters in Rats Exposed to Cadmium.” Biotechnic & Histochemistry 94, no. 5: 381–386. 10.1080/10520295.2019.1574027. 30822167

[fsn370029-bib-0024] Đurendić‐Brenesel, M. , T. Popović , V. Pilija , et al. 2013. “Hypolipidemic and Antioxidant Effects of Buckwheat Leaf and Flower Mixture in Hyperlipidemic Rats.” Bosnian Journal of Basic Medical Sciences 13, no. 2: 100. 10.17305/bjbms.2013.2389. 23725506 PMC4333929

[fsn370029-bib-0025] El‐Kherbawy, G. M. , E. S. Ibrahem , and S. A. Zaki . 2011. “Effects of Parsley and Coriander Leaves on Hypercholesterolemic Rats.”

[fsn370029-bib-0112] Endrini, S. , F. I. A. Bakar , M. F. A. Bakar , N. Abdullah , and H. Marsiati . 2023. “Phytochemical Profiling, In Vitro and In Vivo Xanthine Oxidase Inhibition and Antihyperuricemic Activity of *Christia vespertilionis* Leaf.” Biocatalysis and Agricultural Biotechnology 48: 102645. 10.1016/j.bcab.2023.102645.

[fsn370029-bib-0026] Fahim, M. A. , A. Nemmar , S. Dhanasekaran , et al. 2012. “Acute Cadmium Exposure Causes Systemic and Thromboembolic Events in Mice.” Physiological Research 61, no. 1: 73–80. 10.33549/physiolres.932238. 22188109

[fsn370029-bib-0027] Fahmy, H. A. , N. H. Shreif , and O. A. Gharib . 2014. “The Protective Effect of Coriandium Sativum Extract on Hepato‐Renal Toxicity Induced in Irradiated Rats.” European Journal of Medicinal Plants 4, no. 2: 196. 10.9734/EJMP/2014/7238.

[fsn370029-bib-0028] Farag, M. R. , A. A. Moselhy , A. El‐Mleeh , et al. 2021. “Quercetin Alleviates the Immunotoxic Impact Mediated by Oxidative Stress and Inflammation Induced by Doxorubicin Exposure in Rats.” Antioxidants 10, no. 12: 1906. 10.3390/antiox10121906. 34943009 PMC8750303

[fsn370029-bib-0029] Frankel, E. N. , J. B. German , J. E. Kinsella , E. Parks , and J. Kanner . 1993. “Inhibition of Oxidation of Human Low‐Density Lipoprotein by Phenolic Substances in Red Wine.” Lancet 341, no. 8843: 454–457. 10.1016/0140-6736(93)90206-v. 8094487

[fsn370029-bib-0030] Frydrych, A. , M. Krośniak , and K. Jurowski . 2023. “The Role of Chosen Essential Elements (Zn, cu, se, Fe, Mn) in Food for Special Medical Purposes (FSMPs) Dedicated to Oncology Patients—Critical Review: State‐Of‐The‐Art.” Nutrients 15, no. 4: 1012. 10.3390/nu15041012. 36839370 PMC9961387

[fsn370029-bib-0111] García‐Nieto, V. M. , F. Claverie‐Martín , T. Moraleda‐Mesa , et al. 2022. “Gout Associated with Reduced Renal Excretion of Uric Acid. Renal Tubular Disorder That Nephrologists Do Not Treat.” Nefrología (English Edition) 42, no. 3: 273–279. 10.1016/j.nefroe.2022.05.007. 36210617

[fsn370029-bib-0031] Ghafarifarsani, H. , S. H. Hoseinifar , S. Javahery , M. Yazici , and H. Van Doan . 2022. “Growth Performance, Biochemical Parameters, and Digestive Enzymes in Common Carp ( *Cyprinus carpio* ) fed Experimental Diets Supplemented With Vitamin C, Thyme Essential Oil, and Quercetin.” Italian Journal of Animal Science 21, no. 1: 291–302. 10.1080/1828051X.2021.1965923.

[fsn370029-bib-0032] Glore, S. R. , D. Van Treeck , A. W. Knehans , and M. Guild . 1994. “Soluble Fiber and Serum Lipids: A Literature Review.” Journal of the American Dietetic Association 94, no. 4: 425–436. 10.1016/0002-8223(94)90099-x. 8144811

[fsn370029-bib-0033] Gnoni, G. V. , G. Paglialonga , and L. Siculella . 2009. “Quercetin Inhibits Fatty Acid and Triacylglycerol Synthesis in Rat‐Liver Cells.” European Journal of Clinical Investigation 39, no. 9: 761–768. 10.1111/j.1365-2362.2009.02167.x125. 19508303

[fsn370029-bib-0034] Gordon, D. T. 1992. “The Importance of Total Dietary Fiber in Human Nutrition and Health.” Journal of the Korean Society of Nutrition 25, no. 1: 75–76.

[fsn370029-bib-0035] Gruendel, S. , A. L. Garcia , B. Otto , et al. 2006. “Carob Pulp Preparation Rich in Insoluble Dietary Fiber and Polyphenols Enhances Lipid Oxidation and Lowers Postprandial Acylated Ghrelin in Humans.” Journal of Nutrition 136, no. 6: 1533–1538. 16702317 10.1093/jn/136.6.1533

[fsn370029-bib-0036] Hashim, M. S. , S. Lincy , V. Remya , M. Teena , and L. Anila . 2005. “Effect of Polyphenolic Compounds From *Coriandrum Sativum* on H_2_O_2_‐Induced Oxidative Stress in Human Lymphocytes.” Food Chemistry 92, no. 4: 653–660. 10.1016/j.foodchem.2004.08.027.

[fsn370029-bib-0037] Henry, D. C. , R. S. Neil , and J. S. William . 2003. “Dietary Supplement for Promoting Removal of Heavy Metals From the Body.”

[fsn370029-bib-0038] Hernandez, F. , J. Madrid , V. Garcia , J. Orengo , and M. D. Megias . 2004. “Influence of Two Plant Extracts on Broilers Performance, Digestibility, and Digestive Organ Size.” Poultry Science 83, no. 2: 169–174. 10.1093/ps/83.2.169. 14979566

[fsn370029-bib-0039] Hu, Q. H. , C. Wang , J. M. Li , D. M. Zhang , and L. D. Kong . 2009. “Allopurinol, Rutin, and Quercetin Attenuate Hyperuricemia and Renal Dysfunction in Rats Induced by Fructose Intake: Renal Organic Ion Transporter Involvement.” American Journal of Physiology. Renal Physiology 297, no. 4: F1080–F1091. 10.1152/ajprenal.90767.2008. 19605544

[fsn370029-bib-0040] Hussain, A. , M. R. Arif , A. Ahmed , et al. 2024. “Evaluation of Leaves, Flowers, and Seeds of Coriander (*Coriandrum sativum* L.) Through Microwave Drying and Ultrasonic‐Assisted Extraction, for Biologically Active Components.” Journal of Food Processing and Preservation 2024, no. 1: 2378604. 10.1155/2024/2378604.

[fsn370029-bib-0041] Hussain, A. , T. Kausar , M. A. Jamil , et al. 2022. “In Vitro Role of Pumpkin Parts as Pharma‐Foods: Antihyperglycemic and Antihyperlipidemic Activities of Pumpkin Peel, Flesh, and Seed Powders, in Alloxan‐Induced Diabetic Rats.” International Journal of Food Science 2022, no. 1: 4804408. 10.1155/2022/4804408. 35959224 PMC9363229

[fsn370029-bib-0042] Hussain, A. , S. A. Korma , K. Kabir , et al. 2024. “In Vitro and In Vivo Determination of Biological Activities of Bitter Gourd (*Momordica charantia* L.) Peel, Flesh and Seeds.” Plant Foods for Human Nutrition 79, no. 2: 316–321. 10.1007/s11130-024-01153-2. 38358638

[fsn370029-bib-0043] Hwang, G. H. , Y. R. Heo , H. J. Lee , O. J. Park , S. K. Kang , and Y. D. Kim . 2001. “Effects of *Coriandrum sativum* L. on Lipid Metabolism in Rats With Hypertriglyceridemic Diet.” Nutritional Sciences 4, no. 1: 13–19.

[fsn370029-bib-0044] Iyayi, E. A. , and O. O. Tewe . 1998. “Serum Total Protein, Urea and Creatinine Levels as Indices of Quality of Cassava Diets for Pigs.” Tropical Veterinarian 16: 59–67.

[fsn370029-bib-0045] Kamel, C. 2000. “Natural Plant Extracts: Classical Remedies Bring Modern Animal Production Solutions.” In Proceedings of the III Conference of Feed Manufacturers of the Mediterranean: Feed Manufacturing in the Mediterranean Region Improving Safety: From Feed to Food, edited by J. Brufau , 31–38. Institut Agronomique Mediterraneen de Zaragoza.

[fsn370029-bib-0046] Kaneko, J. J. 1989. Clinical Biochemistry of Domestic Animal. 4th ed. San Diego: Academic Press.

[fsn370029-bib-0047] Karuppasamy, R. , S. Subathra , and S. Puvaneswari . 2005. “Haematological Responses to Exposure to Sublethal Concentration of Cadmium in Air Breathing Fish, *Channa punctatus* (Bloch).” Journal of Environmental Biology 26, no. 1: 123–128. 16114472

[fsn370029-bib-0048] Kenelly, P. J. , K. M. Botham , O. P. McGuinness , V. W. Rodwell , and P. A. Weil . 2018. Illustrated Biochemistry. Vol. 118. 31st ed. Mc Graw Hill. 10.1161/CIRCRESAHA.

[fsn370029-bib-0049] Khalil, S. R. , Y. Abd Elhakim , A. H. Abd El‐fattah , M. R. Farag , N. E. Abd El‐Hameed , and E. M. Abd Elhakeem . 2020. “Dual Immunological and Oxidative Responses in *Oreochromis niloticus* Fish Exposed to Lambda Cyhalothrin and Concurrently Fed With Thyme Powder (*Thymus vulgaris* L.): Stress and Immune Encoding Gene Expression.” Fish & Shellfish Immunology 100: 208–218. 10.1016/j.fsi.2020.03.009. 32165248

[fsn370029-bib-0114] Kim, H. J. 2023. “Antioxidant and XO Inhibitory Activity of Onion Extract in Hyperuricemia Rats.” Journal of Food Biochemistry 47, no. 4: e14280.

[fsn370029-bib-0051] Kozłowska, M. , I. Ścibisz , J. Przybył , M. Ziarno , A. Żbikowska , and E. Majewska . 2021. “Phenolic Contents and Antioxidant Act Scandarivity of Extracts of Selected Fresh and Dried Herbal Materials.” Polish Journal of Food And Nutrition Sciences 71, no. 3: 269–278. 10.31883/pjfns/139035.

[fsn370029-bib-0052] Kulik, K. , I. Kwiecień , B. Chełstowska , E. Rutkowska , and P. Rzepecki . 2021. “Evaluation and Comparison of the New Mindray BC‐6200 Hematology Analyzer With ADVIA 2120i.” International Journal of Laboratory Hematology 43, no. 3: 395–402. 10.1111/ijlh.13418. 33270987

[fsn370029-bib-0053] Kumar Mishra, S. , P. Singh , and S. K. Rath . 2013. “Protective Effect of Quercetin on Chloroquine‐Induced Oxidative Stress and Hepatotoxicity in Mice.” Malaria Research and Treatment 2013: 1–10. 10.1155/2013/141734. PMC3625570 23607047

[fsn370029-bib-0054] Kumar, V. , S. Sood , K. Vasudevan , and G. Umapathy . 2021. “A Practical Method for Storage, Preservation and Transportation of Anuran Urine Samples Using Filter Paper for Hormone Analysis.” MethodsX 8: 101578. 10.1016/j.mex.2021.101578. 35004212 PMC8720910

[fsn370029-bib-0055] Lee, K. W. 2002. “Essential Oils in Broiler Nutrition.” Doctoral diss., Uttercht University. 10.3923/ijps.2004.738.752.

[fsn370029-bib-0119] Lee, K. H. , Y. Kim , E. Park , and H. J. Hwang . 2008. “Effect of Onion Powder Supplementation on Lipid Metabolism in High Fat‐Cholesterol fed SD Rats.” Journal of Food Science and Nutrition 13, no. 2: 71–76.

[fsn370029-bib-0056] Randie, L. 2016. “Laboratory Procedure Manual, Roche Cobas C311 2017—Standard. Fasting Glucose NHANES, 2015–2016.”

[fsn370029-bib-0057] Liu, H. N. , Y. Liu , L. L. Hu , et al. 2014. “Effects of Dietary Supplementation of Quercetin on Performance, Egg Quality, Cecal Microflora Populations, and Antioxidant Status in Laying Hens.” Poultry Science 93, no. 2: 347–353. 10.3382/ps.2013-03225. 24570456

[fsn370029-bib-0058] Liu, Y. , L. Chi , L. Feng , et al. 2011. “Effects of Graded Levels of Dietary Vitamin C on the Growth, Digestive Capacity and Intestinal Microflora of Juvenile Jian Carp ( *Cyprinus carpio* Var. Jian).” Aquaculture Research 42, no. 4: 534–548. 10.1111/j.1365-2109.2010.02649.x.

[fsn370029-bib-0059] Liu, Y. , Y. Li , H. N. Liu , et al. 2013. “Effect of Quercetin on Performance and Egg Quality During the Late Laying Period of Hens.” British Poultry Science 54, no. 4: 510–514. 10.1080/00071668.2013.799758. 23906219

[fsn370029-bib-0060] Lubawy, M. , and D. Formanowicz . 2023. “High‐Fructose Diet–Induced Hyperuricemia Accompanying Metabolic Syndrome–Mechanisms and Dietary Therapy Proposals.” International Journal of Environmental Research and Public Health 20, no. 4: 3596. 10.3390/ijerph20043596. 36834291 PMC9960726

[fsn370029-bib-0062] Mechchate, H. , I. Es‐Safi , A. Amaghnouje , et al. 2021. “Antioxidant, Anti‐Inflammatory and Antidiabetic Proprieties of LC‐MS/MS Identified Polyphenols From Coriander Seeds.” Molecules 26, no. 2: 487. 10.3390/molecules26020487. 33477662 PMC7831938

[fsn370029-bib-0063] Metzler, B. , E. Bauer , and R. Mosenthin . 2005. “Microflora Management in the Gastrointestinal Tract of Piglets.” Asian‐Australasian Journal of Animal Sciences 18, no. 9: 1353–1362. 10.5713/ajas.2005.1353.

[fsn370029-bib-0065] Mo, S. F. , F. Zhou , Y. Z. Lv , Q. H. Hu , D. M. Zhang , and L. D. Kong . 2007. “Hypouricemic Action of Selected Flavonoids in Mice: Structure–Activity Relationships.” Biological and Pharmaceutical Bulletin 30, no. 8: 1551–1556. 10.1248/bpb.30.1551. 17666819

[fsn370029-bib-0066] Moharib, S. A. , and R. S. Adly . 2024. “Hypoglycemic and Hepatoprotective Activities of Coriander (*Coriandrum Sativum*) Extract in Streptozocin Induced Diabetic Rats.” Journal of Advances in Biology & Biotechnology 27, no. 2: 15–38. 10.9734/jabb/2024/v27i2696.

[fsn370029-bib-0067] Mouhoubi, K. , L. Boulekbache‐Makhlouf , K. Madani , M. L. Freidja , A. M. Silva , and S. M. Cardoso . 2023. “Microwave‐Assisted Extraction Optimization and Conventional Extraction of Phenolic Compounds From Coriander Leaves: UHPLC Characterization and Antioxidant Activity.” North African Journal of Food and Nutrition Research 7, no. 15: 69–83. 10.51745/najfnr.7.15.69-83.

[fsn370029-bib-0068] Mountzouris, K. C. , P. Tsirtsikos , E. Kalamara , S. Nitsch , G. Schatzmayr , and K. Fegeros . 2007. “Evaluation of the Efficacy of a Probiotic Containing Lactobacillus, Bifidobacterium, Enterococcus, and Pediococcus Strains in Promoting Broiler Performance and Modulating Cecal Microflora Composition and Metabolic Activities.” Poultry Science 86, no. 2: 309–317. 10.1093/ps/86.2.309. 17234844

[fsn370029-bib-0069] Moustafa, A. H. A. , E. M. M. Ali , S. S. Moselhey , E. Tousson , and K. S. El‐Said . 2014. “Effect of Coriander on Thioacetamide‐Induced Hepatotoxicity in Rats.” Toxicology and Industrial Health 30, no. 7: 621–629. 10.1177/0748233712462470. 23042592

[fsn370029-bib-0070] Nambiar, V. S. , M. Daniel , and P. Guin . 2010. “Characterization of Polyphenols From Coriander Leaves (*Coriandrum sativum*), red Amaranthus (*A. paniculatus*) and Green Amaranthus (*A. frumentaceus*) Using Paper Chromatography and Their Health Implications.” Journal of Herbal Medicine and Toxicology 4, no. 1: 173–177.

[fsn370029-bib-0071] Natesh, H. N. , L. Abbey , and S. K. Asiedu . 2017. “An Overview of Nutritional and Antinutritional Factors in Green Leafy Vegetables.” Horticulture International Journal 1, no. 2: 58–65.

[fsn370029-bib-0072] National Research Council . 2001. Nutrient Requirements of Dairy Cattle: 2001. National Academies Press.

[fsn370029-bib-0073] Nisa, M. U. , M. A. Khan , M. Sarwar , et al. 2006. “Influence of Corn Steep Liquor on Feeding Value of Urea Treated Wheat Straw in Buffaloes Fed at Restricted Diets.” Asian‐Australasian Journal of Animal Sciences 19, no. 11: 1610–1616. 10.5713/ajas.2006.1610.

[fsn370029-bib-0074] Nworgu, F. C. , S. A. Ogungbenro , and K. S. Solesi . 2007. “Performance and Some Blood Chemistry Indices of Broiler Chicken Served Fluted Pumpkin (Telfaria Occidentalis) Leaves Extract Supplement.” American‐Eurasian Journal of Agriculture and Environmental Science 2, no. 1: 90–98.

[fsn370029-bib-0075] Olufemi, B. E. , I. E. Assiak , G. O. Ayoadi , and M. A. Onigemo . 2003. “Studies on Effects of *Amaranthus Spinosus* Leaf Extract on the Haematology of Growing Pigs.” African Journal of Biomedical Research 6, no. 3: 149–150. 10.4314/ajbr.v6i3.54045.

[fsn370029-bib-0076] Pandey, A. , P. Bigoniya , V. Raj , and K. K. Patel . 2011. “Pharmacological Screening of *Coriandrum sativum* Linn. For Hepatoprotective Activity.” Journal of Pharmacy & Bioallied Sciences 3, no. 3: 435–441. 10.4103/0975-7406.84462. 21966166 PMC3178952

[fsn370029-bib-0113] Punzi, L. 2022. “Natural Dietary Antioxidants in Hyperuricemia and Gout Management: The Potential of Flavonoids.” Current Rheumatology Reports 24, no. 3: 70–85.

[fsn370029-bib-0078] Qureshi, A. A. , J. C. Reis , N. Qureshi , C. J. Papasian , D. C. Morrison , and D. M. Schaefer . 2011. “δ‐Tocotrienol and Quercetin Reduce Serum Levels of Nitric Oxide and Lipid Parameters in Female Chickens.” Lipids in Health and Disease 10: 1–22. 10.1186/1476-511X-10-39. PMC3053241 21356098

[fsn370029-bib-0079] Rafique, H. S. , A. Hussain , M. Nadeem , et al. 2023. “Impact of Different Proportions of Sweet Potato (*Ipomoea batatas* L.) Flour on Physical, Chemical and Sensory Parameters of Straight Grade Flour‐Based Cake Rusk.” Food and Humanity 1: 1282–1296. 10.1016/j.foohum.2023.09.024.

[fsn370029-bib-0080] Rafiu, T. A. , O. A. Aderinola , A. O. Akinwumi , T. A. Alabi , and M. D. Shittu . 2013. “Performance and Blood Chemistry of Broiler Chickens Fed *Moringa oleifera* Leaf Meal.” In Proceedings of the 18th Annual Conference of Animal Science Association of Nigeria, 294, 46–52. 10.1590/1806-9061-2016-0373.

[fsn370029-bib-0081] Rahim, M. A. , A. Yasmin , M. Imran , et al. 2022. “Optimization of the Ultrasound Operating Conditions for Extraction and Quantification of Fructooligosaccharides From Garlic (*Allium sativum* L.) via High‐Performance Liquid Chromatography With Refractive Index Detector.” Molecules 27, no. 19: 6388. 10.3390/molecules27196388. 36234922 PMC9573205

[fsn370029-bib-0082] Rahmat, A. , N. S. S. Ahmad , and N. S. Ramli . 2019. “Parsley (*Petroselinum crispum*) Supplementation Attenuates Serum Uric Acid Level and Improves Liver and Kidney Structures in Oxonate‐Induced Hyperuricemic Rats.” Oriental Pharmacy and Experimental Medicine 19: 393–401. 10.1007/s13596-018-0353-7.

[fsn370029-bib-0083] Rajeshwari, U. , and B. Andallu . 2011. “Medicinal Benefits of Coriander (*Coriandrum sativum* L).” Spatula DD 1, no. 1: 51–58. 10.5455/spatula.20110106123153.

[fsn370029-bib-0084] Reeves, P. G. , F. H. Nielsen , and G. C. Fahey Jr. 1993. “AIN‐93 Purified Diets for Laboratory Rodents: Final Report of the American Institute of Nutrition Ad Hoc Writing Committee on the Reformulation of the AIN‐76A Rodent Diet.” Journal of Nutrition 123, no. 11: 1939–1951. 10.1093/jn/123.11.1939. 8229312

[fsn370029-bib-0085] Scandar, S. , C. Zadra , and M. C. Marcotullio . 2023. “Coriander (*Coriandrum sativum*) Polyphenols and Their Nutraceutical Value Against Obesity and Metabolic Syndrome.” Molecules 28, no. 10: 4187. 10.3390/molecules28104187. 37241925 PMC10220854

[fsn370029-bib-0086] Selvakumar, K. , S. Bavithra , S. Suganya , F. Ahmad Bhat , G. Krishnamoorthy , and J. Arunakaran . 2013. “Effect of Quercetin on Haematobiochemical and Histological Changes in the Liver of Polychlorined Biphenyls‐Induced Adult Male Wistar Rats.” Journal of Biomarkers 2013: 1–12. 10.1155/2013/960125. PMC4437364 26317025

[fsn370029-bib-0087] Shahwar, M. K. , A. H. El‐Ghorab , F. M. Anjum , M. S. Butt , S. Hussain , and M. Nadeem . 2012. “Characterization of Coriander (*Coriandrum sativum* L.) Seeds and Leaves: Volatile and Non Volatilenon‐volatile Extracts.” International Journal of Food Properties 15, no. 4: 736–747. 10.1080/10942912.2010.500068.

[fsn370029-bib-0088] Shi, H. T. , S. L. Li , Z. J. Cao , Y. J. Wang , G. M. Alugongo , and P. H. Doane . 2015. “Effects of Replacing Wild Rye, Corn Silage, or Corn Grain With CaO‐Treated Corn Stover and Dried Distillers Grains With Solubles in Lactating Cow Diets on Performance, Digestibility, and Profitability.” Journal of Dairy Science 98, no. 10: 7183–7193. 10.3168/jds.2014-9273. 26210280

[fsn370029-bib-0089] Shin, J. W. , I. C. Seol , and C. G. Son . 2010. “Interpretation of Animal Dose and Human Equivalent Dose for Drug Development.” Journal of Korean Medicine 31, no. 3: 1–7.

[fsn370029-bib-0090] Shoko, T. , V. E. Manhivi , M. Mtlhako , and D. Sivakumar . 2022. “Changes in Functional Compounds, Volatiles, and Antioxidant Properties of Culinary Herb Coriander Leaves (*Coriandrum sativum*) Stored Under Red and Blue LED Light for Different Storage Times.” Frontiers in Nutrition 9: 856484. 35634386 10.3389/fnut.2022.856484 PMC9134111

[fsn370029-bib-0091] Soliman, M. M. , M. A. Nassan , A. Aldhahrani , F. Althobaiti , and W. A. Mohamed . 2020. “Molecular and Histopathological Study on the Ameliorative Impacts of Petroselinum Crispum and *Apium graveolens* Against Experimental Hyperuricemia.” Scientific Reports 10, no. 1: 9512. 10.1038/s41598-020-66205-4. 32528050 PMC7289838

[fsn370029-bib-0092] Sorial, A. M. 2021. “Hypolipidemic Activities and Nutritive Values of Brassica Napus and *Eruca Sativa* Seed Supplementation in Rats Fed a High Cholesterol Diet.” EC Veterinary Science 6: 29–40.

[fsn370029-bib-0093] Steel, R. 1997. “Analysis of Variance I: The One‐Way Classification.” In Principles and Procedures of Statistics – A Biometrical Approach, 139–203. McGraw‐Hill3rd ed.

[fsn370029-bib-0094] Su, H. Y. , C. Yang , D. Liang , and H. F. Liu . 2020. “Research Advances in the Mechanisms of Hyperuricemia‐Induced Renal Injury.” BioMed Research International 2020, no. 1: 5817348. 10.1155/2020/5817348. 32685502 PMC7336201

[fsn370029-bib-0095] Sukhotnik, I. , D. Moati , R. Shaoul , B. Loberman , Y. Pollak , and B. Schwartz . 2018. “Quercetin Prevents Small Intestinal Damage and Enhances Intestinal Recovery During Methotrexate‐Induced Intestinal Mucositis of Rats.” Food & Nutrition Research 62: 1327. 10.29219/fnr.v62.1327. PMC5883860 30026677

[fsn370029-bib-0096] Tang, E. L. , J. Rajarajeswaran , S. Y. Fung , and M. S. Kanthimathi . 2013. “Antioxidant Activity of *Coriandrum sativum* and Protection Against DNA Damage and Cancer Cell Migration.” BMC Complementary and Alternative Medicine 13, no. 1: 1–13. 10.1186/1472-6882-13-347. 24517259 PMC4028854

[fsn370029-bib-0097] Tebib, K. , L. Bitri , P. Besançon , and J. M. Rouanet . 1994. “Polymeric Grape Seed Tannins Prevent Plasma Cholesterol Changes in High‐Cholesterol‐Fed Rats.” Food Chemistry 49, no. 4: 403–406. 10.1016/0308-8146(94)90012-4.

[fsn370029-bib-0098] Teirlynck, E. , L. Bjerrum , V. Eeckhaut , et al. 2009. “The Cereal Type in Feed Influences Gut Wall Morphology and Intestinal Immune Cell Infiltration in Broiler Chickens.” British Journal of Nutrition 102, no. 10: 1453–1461. 10.1017/S0007114509990407. 19664304

[fsn370029-bib-0099] Ufele, A. N. , B. O. Mgbenka , and J. F. Ude . 2008. “Effect of Nutrition on the Red Blood Cells of Trypanosome‐Infected Female Rats.” Animal Research International 5, no. 1: 816–818. 10.4314/ari.v5i1.48720.

[fsn370029-bib-0118] Umer, M. , M. U. Nisa , N. Ahmad , M. A. Rahim , and F. Al‐Asmari . 2023. “Effects of Different Levels of Dried Onion Powder on Nutrient Digestibility, Biochemical Parameters, and Nitrogen Balance in Wistar Albino Rats with Induced Hyperuricemia.” Frontiers in Physiology 14: 1273286. 10.3389/fphys.2023.1273286. 38111897 PMC10725973

[fsn370029-bib-0117] Umer, M. , M. U. Nisa , N. Ahmad , M. A. Rahim , and L. M. Kasankala . 2024. “Quantification of Quercetin from Red Onion (*Allium cepa* L.) Powder via High‐Performance Liquid Chromatography‐Ultraviolet (HPLC‐UV) and Its Effect on Hyperuricemia in Male Healthy Wistar Albino Rats.” Food Science & Nutrition 12, no. 2: 1067–1081. 38370075 10.1002/fsn3.3822 PMC10867493

[fsn370029-bib-0102] Verma, N. , and N. Trehan . 2013. “HPLC Analysis of Methanolic Extract of Herbs for Quercetin Content.” Journal of Pharmacognosy and Phytochemistry 2, no. 1: 159–162.

[fsn370029-bib-0103] Wade, N. 1978. “NIH Considers Animal Rights.” Science 199, no. 4326: 279. 10.1126/science.199.4326.279.a. 17759651

[fsn370029-bib-0104] Wang, M. I. , F. L. Xiao , Y. J. Mao , L. L. Ying , B. Zhou , and Y. Li . 2019. “Quercetin Decreases the Triglyceride Content Through the PPAR Signalling Pathway in Primary Hepatocytes of Broiler Chickens.” Biotechnology & Biotechnological Equipment 33, no. 1: 1000–1010. 10.1080/13102818.2019.1635528.

[fsn370029-bib-0105] Zengin, G. , A. Aktumsek , G. O. Guler , Y. S. Cakmak , and E. Yildiztugay . 2011. “Antioxidant Properties of Methanolic Extract and Fatty Acid Composition of Centaurea Urvillei DC. subsp. Hayekiana Wagenitz.” Records of Natural Products 5, no. 2: 123–132.

[fsn370029-bib-0106] Zhang, S. , and I. H. Kim . 2020. “Effect of Quercetin (Flavonoid) Supplementation on Growth Performance, Meat Stability, and Immunological Response in Broiler Chickens.” Livestock Science 242, no. 104: 286. 10.1016/j.livsci.2020.104286.

[fsn370029-bib-0107] Zhang, Y. , H. Li , Y. Zhao , and Z. Gao . 2006. “Dietary Supplementation of Baicalin and Quercetin Attenuates Iron Overload Induced Mouse Liver Injury.” European Journal of Pharmacology 535, no. 1–3: 263–269. 10.1016/j.ejphar.2006.01.067. 16527270

